# Bayesian uncertainty quantification to identify population level vaccine hesitancy behaviours

**DOI:** 10.1371/journal.pone.0349499

**Published:** 2026-05-26

**Authors:** David J. Warne, Abhishek Varghese, Aidan Brewster, Alexander P. Browning, Mario M. Krell, Christopher Drovandi, Wenbiao Hu, Antonietta Mira, Kerrie Mengersen, Adrianne L. Jenner

**Affiliations:** 1 School of Mathematical Sciences, Queensland University of Technology, Brisbane, Queensland, Australia; 2 Centre for Data Science, Queensland University of Technology, Brisbane, Queensland, Australia; 3 Mathematical Institute, University of Oxford, Oxford, United Kingdom; 4 School of Mathematics and Statistics, University of Melbourne, Melbourne, Victoria, Australia; 5 Graphcore Inc., Palo Alto, California, United States of America; 6 School of Public Health and Social Work, Queensland University of Technology, Brisbane, Queensland, Australia; 7 Faculty of Economics, Universitá della Svizzera Italiana, Lugano, Switzerland; 8 Department of Science and High Technology, Insubria University, Varese, Italy; The University of New Mexico, UNITED STATES OF AMERICA

## Abstract

When effective vaccines are available, vaccination programs are typically one of the best defences against the spread of an infectious disease. Such vaccination programs become particularly important during severe epidemics or pandemics to ensure sufficient vaccination coverage is achieved to increase protection or reduce transmission to a level that enables relaxation of non-pharmaceutical interventions, such as lockdowns, travel restrictions, or social distancing. Unfortunately, vaccination uptake in the community may be slow if there are substantial levels of vaccine hesitancy in the population. As a result, it is important to identify when these hesitancy behaviours are present in the community. Furthermore, understanding the main drivers of such behaviour can inform adjustments to public health strategies to improve community uptake. In this study, we consider the problem of identifying vaccination hesitancy behaviour during a vaccination roll-out that occurs in response to a severe epidemic. Specifically, our aim is to explore the extent to which mathematical modelling of reported case, death, and vaccination counts can be used to detect vaccine hesitancy and possible drivers. To do this, we develop a novel susceptible-exposed-infectious-recovered (SEIR) epidemiological model of disease transmission that incorporates changes in population behaviour relating to non-pharmaceutical interventions and vaccine uptake that are influenced by information reported through media or data dashboards about cases, deaths, and vaccination rates. We then use a Bayesian approach to analyse simulated data representing various hesitancy scenarios. Through this simulation study, our key findings are that individual parameters values related to drivers of vaccine hesitancy often cannot be identified. However, posterior correlation structures between these parameters enable the presence of vaccine hesitancy in the community to be detected and provide some insight into the relative influence of key factors, such as vaccine safety concerns or complacency. While our simulation study is inspired by the public health response to the COVID-19 pandemic, our tools and techniques are general and could enable vaccination programs of various infectious diseases to be adapted rapidly in response to community behaviours in the future.

## Introduction

To reduce transmission and mortality due to a vaccine-preventable infectious disease, a certain level of vaccination coverage is needed to ensure a sufficient level of protection or reduction in transmission such that non-pharmaceutical interventions (NPIs) can be relaxed. Depending on the specific disease, this vaccination coverage target may be related to achieving a certain level of protection via herd immunity, or in other cases, the vaccination target may be related to reduction in transmission as for endemic diseases with waning immunity and emerging variants. Regardless of the specific vaccination mechanism that enables relaxation of NPIs, both the supply and demand for the vaccine must be sufficiently high to maintain a large enough vaccination uptake to reach the required vaccination target. The factors contributing to the success of a vaccination program within a particular community are complex and must be assessed within the cultural and societal context of the program [1,2].

One particular concern within a vaccination program is that of *vaccine hesitancy*, that is, the delay in acceptance or refusal of vaccination despite availability of vaccination services [3–5]. The so-called 3C model [4] of vaccine hesitancy [2] identifies a variety of factors that influence vaccine hesitancy within the categories of *complacency*, *convenience*, and *confidence*. In this setting, complacency refers to the low perceived risk of a vaccine-preventable disease, convenience refers to the ease of getting vaccinated, and confidence refers to the trust in vaccine efficacy and safety, including trust in the healthcare system more broadly [4]. The different effects of vaccination are important to consider when it comes to vaccine confidence. For example, a vaccine may substantially reduce the severity of a disease while having little effect on transmission, and this may lead to reduced community confidence as reported cases continue to increase. While in many cases the majority accept vaccination, there can be context specific reasons why an understanding of vaccine hesitancy patterns within a sub-population is essential to ensure an effective vaccination program [4].

During a severe epidemic or pandemic in which NPIs are necessary control measures, understanding the impact of vaccine hesitancy is crucial to plan the relaxation of restrictions [5,6]. Assuming governments do take appropriate responsibility for decision making [7,8], then identifying vaccine hesitancy patterns in a population is crucial for affirmative action by governments, healthcare institutions and policy-makers to improve vaccine uptake. The goal of this work, is to demonstrate the feasibility of using reported case data to distinguish a slow vaccine uptake due to vaccine hesitancy from limitations in vaccination rates due to supply and logistics. This identification of vaccine hesitancy behaviours in a population could be applied to assist in the design of targeted follow-up studies and policy amendments to address the main area affecting hesitancy.

A wealth of information regarding the driving forces behind vaccine hesitancy can be obtained through statistical machine learning approaches using survey data [9–13]. During a vaccine roll-out these approaches can used to assess the willingness of individuals to get vaccinated [10] or account for effects of media and other information sources on attitudes to vaccination [9,11–14]. While these investigations analysing survey data are insightful in identifying predictors of vaccine hesitancy, there are limitations to the survey approach. In particular, survey-based approaches often rely on detailed data being available, thus limiting the applicability of the model to other countries or jurisdictions [15]. Furthermore, a survey cannot model the effect that disease dynamics has on vaccine hesitancy and *vice versa*. Unfortunately, there have been few mathematical modelling studies that consider this dynamic. Some examples explore the impacts of information on the behaviour related to vaccine hesitancy [16,17] and strategies to counter misinformation [18–20].

Following work by Buonomo [16], Warne et al., [21] and Le et al., [22], we develop mathematical modelling and statistical analysis tools to quantify vaccine hesitancy behaviour patterns during a vaccine roll-out while NPIs are eased. We use synthetically generated reported case numbers that are characteristic of real-world standardised measurements. We incorporate the effect of a single vaccine requiring two doses into a stochastic epidemiological model that accounts for changes in behaviour related to transmission (i.e., related to NPIs and community compliance) and vaccine uptake. Changes in population behaviour are driven by reported disease case data that result in feedback loops. We demonstrate that a variety of realistic dynamics can be produced with our model under different scenarios for NPI strategies, vaccination roll-outs, and hesitancy behaviours. To obtain insight into the identification of vaccine hesitancy behaviours during a real vaccination campaign, the model is calibrated to synthetic data to determine if reported case data are sufficiently informative in relation to vaccine hesitancy [23,24]. In this work, we address this important and often overlooked aspect of mathematical modelling by applying Bayesian uncertainty quantification. We demonstrate through a simulation study that different hesitancy behaviours, such as complacency and vaccine safety concerns can be distinguished from each other using reported case data and vaccination counts. This framework has the potential to assist public health policy makers in identifying the dominating causes of vaccine hesitancy rapidly to complement results of survey data analysis [15].

## Materials and methods

In this section, we present a framework to analyse vaccine hesitancy behaviour patterns during an epidemic. We demonstrate our approach using synthetic data inspired by publicly available case data and vaccination counts that were actively tracked during the COVID-19 pandemic. Our approach consists of a novel stochastic epidemiological model that accounts for changes in transmission driven by behavioural changes related to NPIs and changes in vaccination uptake based on confidence and complacency types of hesitancy behaviour.

### Data sources and structure

We consider a scenario in which time-series of cumulative reported cases, deaths and vaccination doses are actively maintained and monitored during the epidemic. There are a number of challenges in using this type of data source to model the epidemic evolution and understanding hesitancy. Firstly, the cumulative confirmed cases do not necessarily represent the actual number of infections that have occurred in the population. Primarily this will be due to undetected asymptomatic cases [25,26], but could also be impacted to a substantially lesser degree by lower sensitivity or specificity in diagnostic test results. These undetected cases need to be accounted for since asymptomatic cases can still effectively transmit disease [27]. Second, the cumulative case recoveries often needs to be estimated as a proportion of cumulative cases that did not die and are not currently active. Finally, and most importantly for the study of vaccine hesitancy, the cumulative case and death data is often not stratified by vaccination status. While the vaccination status of deaths is recorded by hospitals [28], it is typically not available in large scale aggregated data available from CDC dashboards [29,30]. Therefore the distribution of vaccination status across cases and deaths also needs to be inferred when working with dashboard data.

Since this work is designed to evaluate the potential of our methods to identify hesitancy behaviour patterns, we rely on simulated data using a model with known hesitancy patterns. However, our modelling and simulated data processes are constructed in such a way that they are consistent with real epidemiological data repositories such as those provided by Johns Hopkins University [29] or Our World in Data [30] during the COVID-19 pandemic. For our simulation study, we have selected key population and epidemiological parameters that are qualitatively similar to real scenarios that occurred during the COVID-19 pandemic, however, our various hesitancy scenarios are all hypothetical and no conclusions are claimed in relation to vaccine hesitancy during COVID-19 vaccination roll-outs.

### Model development

Here, we describe our stochastic epidemiological model that incorporates both the effects on NPIs and the effect of vaccination hesitancy. Our approach builds upon the model employed by Warne et al., [21] and Le et al., [22] to quantify community behaviour in relation to NPIs and potential strategies for NPI relaxation. Nomenclature is provided in Tables 1 and 2 for reference throughout theses sections. For full mathematical details of the models see S1 Appendix.

**Table 1:** List of compartments in the full SEIR model including the case reporting process of a two-dose vaccination program. A state is considered observable if it is available in a publicly available repository. States labelled with an asterisk “^*^” indicate data used in reporting, but aggregated before public release.

Symbol	Description	Observable
*P*	Total population of a region	yes
$S_{i}$	Susceptible vaccinated dose i∈{0,1,2}	no
*E*_*i*_	Exposed vaccinated dose i∈{0,1,2}	no
*I*_*i*_	Infectious vaccinated dose i∈{0,1,2}	no
*R*_*i*_	Recovered vaccinated dose i∈{0,1,2}	no
$A_{i}^{*}$	Active confirmed case vaccinated dose i∈{0,1,2}	no
$R_{i}^{*}$	Case recovery vaccinated dose i∈{0,1,2}	no
$D_{i}^{*}$	Case fatality vaccinated dose i∈{0,1,2}	no
*A* ^*^	Total active confirmed cases	yes
*R* ^*^	Total case recoveries	yes
*D* ^*^	Total case fatalities	yes
$V_{i}^{*}$	Total vaccinated population dose i∈{1,2}	yes

**Table 2:** Model parameters fixed for synthetic dataset generation. Parameters related to vaccination rates and hesitancy are estimated via Bayesian inference. All other parameter values are obtained from the literature [21,31].

Symbol	Description	Units	Value
$\alpha_{0}$	Residual transmission rate	[1/days]	0.05
α	Transmission rate	[1/days]	0.4
1/β	mean incubation period	[days]	14
γ	Case identification rate	[1/days]	0.05
ρ	Case recovery rate	[1/days]	0.05
δ	Case death rate	[1/days]	0.03
η	Unobserved recovery rate	[1/days]	0.1
κ	initial ratio infectious to confirmed active cases	–	10
ζ	initial proportion of living cases that are active	–	0.1527
1/ω	mean duration between doses	[days]	21
$\alpha_{v}^{1}$	Transmission reduction with dose 1	–	0.693
$\alpha_{v}^{2}$	Transmission reduction with dose 2	–	0.048
$\delta_{v}^{1}$	Death rate reduction with dose 1	–	0.08
$\delta_{v}^{2}$	Death rate reduction with dose 2	–	0.047
*T*_*d*_	the day that restrictions are lifted	[days]	92
*T*_*v*_	the day that vaccine roll-out begins	[days]	92
*n*	response function g(·) slope parameter	–	10
*n*_*v*_	hesitancy function h(·) slope parameter	–	2
*w*_*A*_	response function active case weight	–	3 × 10^−4^

**Fig 1:**
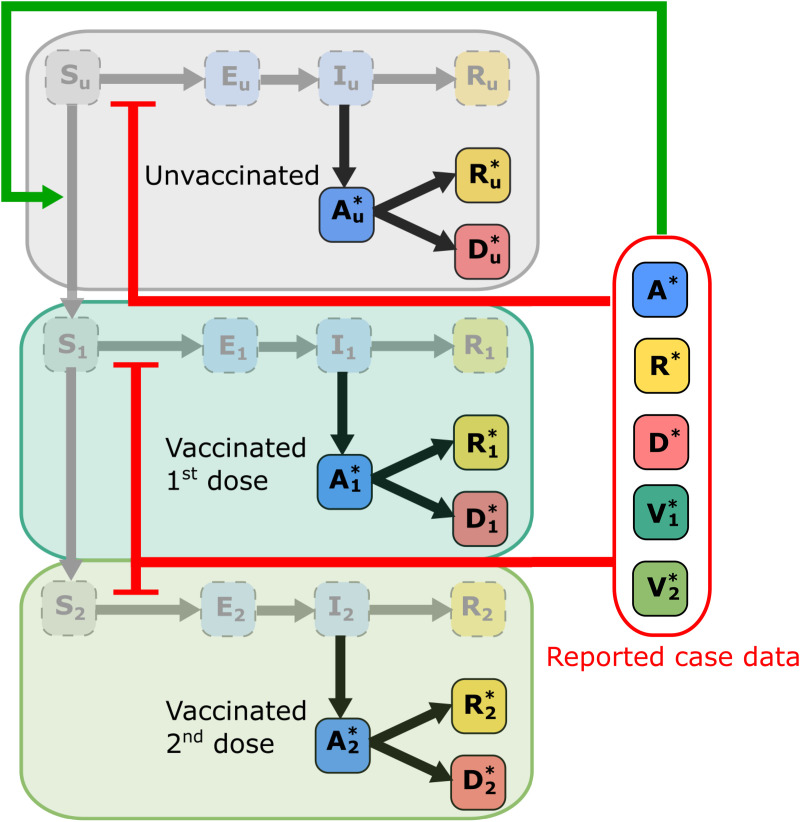
Schematic of the epidemiological model including two-dose vaccination program. States are marked as labelled boxes and arrows indicate state transitions. The model consists of three vaccinations stages, each with its own SEIR model with the reported case data arising through the transition from *I*_*u*_ to $A_{u}^{*}$ (resp. *I*_1_ to $A_{1}^{*}$ and *I*_2_ to $A_{2}^{*}$). The transmission is inhibited by a response function that depends on the aggregated observed reported case data. Similarly, vaccination is promoted through the hesitancy effect function that depends on the aggregated observed reported case data.

Our model is a stochastic susceptible-exposed-infectious-recovered (SEIR) compartmental model. In this framework, a population of size *P* is divided into four main epidemiological compartments: susceptible (*S*), exposed (*E*), infectious (*I*) and recovered (*R*). Since we cannot observe exactly the true number of active infectious individuals, these components are considered unobservable or latent states. We consider the introduction of a vaccination program including a single vaccine-brand with a two dose vaccination protocol (e.g., AstraZeneca or Pfizer only), that is, each compartment is further resolved by vaccination status: unvaccinated, vaccinated (1st dose), and fully vaccinated (2nd dose) (Fig 1).

Since the population counts within each of these compartments are not directly observable in reality, these states are treated as unobservable latent states and we include an observation process based on diagnostic testing in the infectious population. Within the same vaccination status *i*, infectious individuals (*I*_*i*_) will be identified as active confirmed cases ($A_{i}^{*}$) at rate γ>0. These active confirmed cases will transition to reported deaths ($D_{i}^{*}$) or recoveries ($R_{i}^{*}$) at rates $\delta_{i}>0$ and ρ>0 respectively. If we accumulate these observable case numbers over vaccination status, then we obtain three observables: the total confirmed active cases $A^{*}=A_{0}^{*}+A_{1}^{*}+A_{2}^{*}$, total case deaths, $D^{*}=D_{0}^{*}+D_{1}^{*}+D_{2}^{*}$, total case recoveries, $R^{*}=R_{0}^{*}+R_{1}^{*}+R_{2}^{*}$, total vaccinations (1st dose), $V_{1}^{*}=S_{1}+E_{1}+I_{1}+R_{1}+A_{1}^{*}+D_{1}^{*}+R_{1}^{*}$, and total fully vaccinated $V_{2}^{*}=S_{2}+E_{2}+I_{2}+R_{2}+A_{2}^{*}+D_{2}^{*}+R_{2}^{*}$. These observables correspond to reported data available from online dashboards and repositories during the COVID-19 pandemic [29,30]. It should be noted that most data sources only reliably record the cumulative case numbers, $C^{*}=A^{*}+R^{*}+D^{*}$, and reported deaths, D^*^. In addition, the reported C^*^ will be an underestimate of the true number of cumulative cases since infectious individuals can also recover without being reported as a confirmed case. In our model this transition occurs with rate η>0.

We assume vaccination only affects the disease transmission and mortality rate. At each vaccination level i∈{0,1,2}, modifiers $\alpha_{v,i}\in (0,1]$ and $\delta_{v,i}\in (0,1]$ are applied to the baseline transmission rates α>0 and mortality rate δ>0, respectively. For example, at vaccination level *i* the effective mortality rate is $\delta_{v}^{i}\delta$. We set and assume $\alpha_{v}^{2}<\alpha_{v}^{1}<1$ and $\delta_{v}^{2}<\delta_{v}^{1}<1$. Estimates for these modifier parameters would typically be obtained from the literature resulting from vaccination clinical trials [28,31–33]. For the purposes of our simulation study, we derived rough estimates based on literature on the Oxford-AstraZeneca vaccine (ChAdOx1) in relation to the B.1.617.2 (Delta) variant of SARS-CoV-2 virus [31]. There are also vaccination rate parameters, ν>0 and ω>0. Here, ν represents the rate at which individuals get vaccinated in the absence of any vaccine hesitancy, and ω is the rate at which individuals who received their first dose 1/ω days ago will proceed to second dose as per protocol, this is implemented using a delay term (See S1 Appendix). We assume that those currently infected, that is exposed and infectious individuals, do not get vaccinated until after recovery. This simplifies the model so that the only possible transitions between vaccination levels are for the susceptible and recovered compartments, *S*_*i*_, *R*_*i*_, and $R_{i}^{*}$.

**Fig 2:**
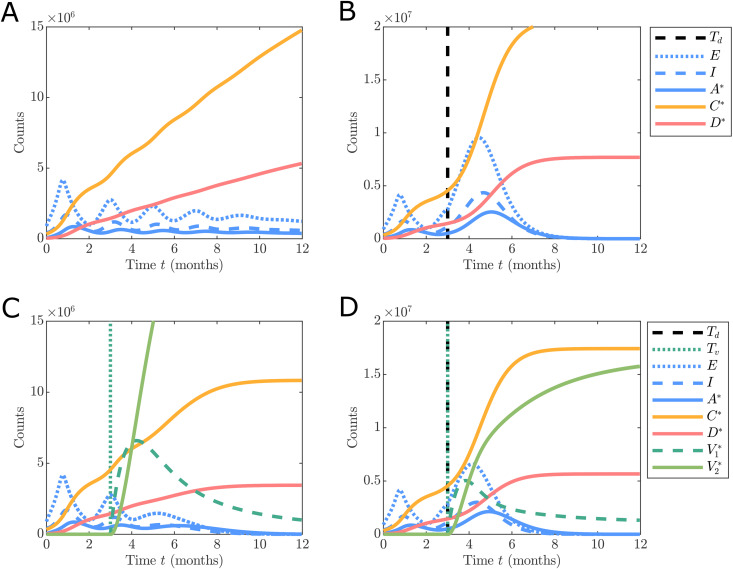
Comparison of the effect of an AstraZeneca-like vaccination program under different configurations of response function and vaccination inclusion (assuming no vaccine hesitancy). Simulations are initialised based on the UK data on 1st September 2020 with $C_{0}^{*}=337,798$, $D_{0}^{*}=41,551$. **(A)** and **(C)**: corresponds to ongoing small lockdowns, **(B)** and **(D)**: corresponds to complete easing of restrictions after *T* ≥ 92 (roughly the 2nd of December 2020). A single stochastic realisation is shown in **(A)**–**(D)** using an approximate stochastic simulation scheme (See S1 Appendix).

Given the large well-mixed populations that we consider here (Fig 2), it is true that deterministic models may provide good approximations for the mean, however there are a number of key reasons we focus on the stochastic setting in this work. Firstly, even though the observable states are large, this does not mean that extinction events or very small populations cannot occur within any single component; ignoring this possibility can have a non-negligible effect on the system dynamics due to the two non-linear feedback mechanisms. Secondly, even when the simulation error is small, this can have an unpredictable effect on parameter inference and uncertainty quantification [34,35]. Finally, we wish to provide a computational framework that is widely applicable to other settings that include smaller populations [21]. While working with a stochastic model comes at a computational cost, we mitigate this computational burden using efficient approximate stochastic simulation and Bayesian inference scheme.

In our model, the virus transmission rate and the vaccination rate are modulated by two feedback mechanisms representing how a population changes their behaviour in response to information related to the observable states $C^{*},D^{*},V_{1}^{*}$ and $V_{2}^{*}$. The first is a negative feedback *response function* g(·)∈[0,1] that affects the transmission rate and represents the implementation of voluntary or mandated NPIs including the compliance thereof (See S1 Appendix for details). Such mechanisms have been explored for a variety of infectious disease to identify media influences [21,22,36,37]. A variety of forms for this response function can be implemented [21], here we employ an NPI implementation strategy based on a trigger threshold,


$$\begin{array}{c} g(A^{*},t)=\frac{1}{1+(w_{A}A^{*}{)}^{n}}1_{\left(0,T_{d}\right]}\left(t\right)+1_{\left(T_{d},\infty \right)}\left(t\right), \end{array}$$ (1)

where *T*_*d*_ > 0 is the point in time that NPIs cease and *w*_*A*_ is a weight parameter setting the number of reported active cases at which NPI efficacy reaches 50%. Note that for *t* > *T*_*d*_ the $g(A^{*},t)=1$. See S1 Appendix for additional details.

Fig 2 demonstrates the effect that vaccination has on the virus spread and severity using example stochastic realisations from our model parameterised by Fig 2 under different scenarios of response behaviour with the assumption no hesitancy behaviour (that is, perfect vaccination rate of ν>0 for $t>T_{v}>0$ where *T*_*v*_ is the start time of the vaccination roll-out). Fig 2(A) shows a continued lockdown using Eq. (1) with $T_{d}\to \infty$ and no vaccination roll-out $T_{v}\to \infty$. Fig 2(B) shows a similar scenario, however with NPIs ceasing at *T*_*d*_ = 3 (months). Comparing Fig 2(A) and 2(B) demonstrates the effect of ceasing NPIs in the absence of any effective vaccine, that is, an order of magnitude more reported cases and deaths. Fig 2(C) and 2(D) show the same lockdown scenarios as Fig 2(A) and 2(B), however, introducing a vaccination roll-out commencing at *T*_*v*_ = 3 (months). We observe, as expected, a reduction in the total case deaths due to vaccination (Compare Fig 2(A) with 2(C) and Fig 2(B) with 2(D)). We also note a chance of elimination occurring when restrictions continue along with the vaccination program (Fig 2(C)), such elimination behaviours can only be captured using a stochastic model.

The second feedback loop relates to the vaccine hesitancy. We denote h(·)∈[0,1] as the *hesitancy effect* function resulting in an effective vaccination rate of νh(·). We assume that vaccine hesitancy behaviour only affects the probability of an individual receiving the first dose with every individual that gets vaccinated continuing to obtain a second dose to be “fully vaccinated”. We note that this assumption may not always be appropriate, especially for diseases requiring boosters or adverse reactions to the first dose reducing the likelihood of an individual returning for the second dose (see Discussion). Just as with the response function, the hesitancy effect function only depends on the observable states and time. We treat h(·) as an increasing function with h(·)=0 corresponding to a situation where individuals refuse vaccination entirely and h(·)=1 leads to no hesitancy effect with the maximum rate ν achieved. This function is intended to model the way in which reported case and vaccination data may increase the probability of an individual seeking vaccination.

We can construct a hesitancy effect function to capture the complacency and confidence components of the 3C model of vaccine hesitancy [4]. We do not consider the convenience component due to the well-mixed assumption (See Discussion). If we consider a population that is only affected by complacency, then it is reasonable to assume the incidence of a disease in a community will tend to increase the likelihood of an individual to seek vaccination [16,18] as the perceived risk increases to overcome complacency. This complacency pattern could be modelled with the function,


$$\begin{array}{c} h(C^{*},D^{*},t)=\frac{(w_{C}C^{*}+w_{D}D^{*}{)}^{n_{v}}}{1+(w_{C}C^{*}+w_{D}D^{*}{)}^{n_{v}}}{𝟙}_{\left[T_{v},\infty \right)}\left(t\right), \end{array}$$ (2)

where $w_{C},w_{D}>0$ are weights reflecting the influence of data reports on population behaviour and *n*_*v*_ > 0 is a slope parameter that governs the sensitivity of the behaviour change to increased incidence, and *T*_*v*_ is that time the vaccine roll-out starts with ${𝟙}_{\left[T_{v},\infty \right)}\left(t\right)=0$ for *t* < *T*_*v*_. The relative importance of case numbers and deaths in influencing an individuals decision to get vaccinated can be assessed by considering the case where $C^{*}=(w_{D}/w_{C})D*$, that is each new death has as much influence as $w_{D}/w_{C}$ new cases. Of course, motivation could be completely dominated by cases (resp. deaths), in which case *w*_*D*_ = 0 (resp. *w*_*C*_ = 0). In practice, due to the strong correlation between total confirmed cases and deaths, it is perfectly reasonable to set $w_{C}=0$.

Another cause of vaccine hesitancy behaviour are vaccine safety concerns, related to confidence in the 3C model [4]. Such safety concerns were prevalent in the COVID-19 pandemic due to rapid development of vaccines [38] and some widely publicised, though rare, side effects [19,20]. If only vaccination safety is a concern, then critical mass of fully vaccinated individuals may be necessary to alleviate these concerns. This can be reflected in the function,


$$\begin{array}{c} h(V_{2}^{*},t)=\frac{(w_{V}V_{2}^{*}{)}^{n_{v}}}{1+(w_{V}V_{2}^{*}{)}^{n_{v}}}{𝟙}_{\left[T_{v},\infty \right)}\left(t\right), \end{array}$$ (3)

with weight parameter *w*_*V*_ > 0. Note that in this case, at time *T*_*v*_ some initial non-zero vaccinated population must be present, otherwise the vaccinated population will remain at $V_{2}^{*}=0$. The initial population could represent participants in relevant clinical trials, or an initial vaccination mandate for particular occupations (e.g., healthcare workers). In reality, other factors beyond vaccine safety could affect confidence especially in situations where the vaccine reduces disease severity without impacting transmission. In this setting, Eq. (3) could be extended to enable continued increase in reported cases to reduce confidence, which would work against the increasing case numbers in the complacency function Eq (2).

Finally, we can consider a population hesitancy behaviour that is due to safety concerns and complacency,


$$\begin{array}{c} h(C^{*},D^{*},V_{2}^{*},t)=\frac{(w_{C}C^{*}+w_{D}D^{*}+w_{V}V_{2}^{*}{)}^{n_{v}}}{1+(w_{C}C^{*}+w_{D}D^{*}+w_{V}V_{2}^{*}{)}^{n_{v}}}{𝟙}_{\left[T_{v},\infty \right)}\left(t\right). \end{array}$$ (4)

Once again the weights $w_{C},w_{D},w_{V}\geq 0$ provide a measure of the relative influence each new case, death or vaccination has on the probability of an individual getting vaccinated. In reality, these parameters will be unknown, however if standard COVID-19 case data can provide insight into the values of these parameters [21], then we can start to assess the trends in hesitancy behaviours for a given population of interest.

### Simulation study

**Fig 3:**
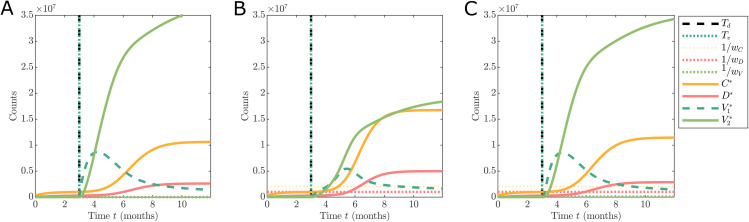
Comparison of different hesitancy effect functions show the dynamics of the observable states. The scenarios are: (A) no hesitancy behaviour with $h(\cdot ,t)={𝟙}_{\left[T_{v},\infty \right)}\left(t\right)$; (B) hesitancy effect driven by complacency only with h(·) defined in Eq. (2); and (C) hesitancy effect driven by mild safety concerns and some complacency only with h(·) defined in Eq. (4). Simulations are initialised based on the UK data on 1st September 2020 with $C_{0}^{*}=337,798$, $D_{0}^{*}=41,551$, and model parameters are given in Table 2 with ν=0.01, *n*_*v*_ = 4, $w_{C}=2\times 10^{-6}$, $w_{D}=1\times 10^{-6}$, and $w_{V}=1\times 10^{-5}$.

Given that reported data are aggregated, it is unclear if the data are informative enough to identify what type of hesitancy effect is occurring in reality. For example, three different scenarios are shown in Fig 3 corresponding to no hesitancy (Fig 3(A)), complacency only (Fig 3(B)), and complacency and vaccine safety concerns (Fig 3(C)). While each evolution is different qualitatively, it is completely unclear if the scenarios including different hesitancy effects can be distinguished from a scenario with no hesitancy effect but slower vaccination rate. For example, such is the difference in the hesitancy behaviour between Fig 3(C) and the no hesitancy case of Fig 3(A), yet the differences in the dynamics are subtle. Here Fig 3(C) looks like Fig 3(A) with a slower vaccination rate, however, in reality both have the same rate and the effect is entirely due to hesitancy.

The behaviour observed in Fig 3 provides motivation to explore the informativity of aggregated reported COVID-19 data as provided by online repositories synthetically for the purpose of analysing vaccine hesitancy behaviour. To explore this, we consider a simulation study and develop Bayesian analysis techniques.

We generate multiple simulated datasets under three different vaccine uptake behaviours. Specifically, we consider: complacency-based hesitancy (*w*_*V*_ = 0, *w*_*C*_ > 0, and *w*_*D*_ > 0), complacency and vaccine safety (*w*_*V*_ > 0, *w*_*C*_ > 0, and *w*_*D*_ > 0) hesitancy, and no hesitancy (h(·)=1). In each of the simulation settings we vary the weights and the maximum vaccination rate, the remaining parameters are fixed based on the literature [21,28,31–33] as listed in Table 2.

### Bayesian analysis

Given a simulated dataset, $𝒟=\{(C_{t}^{*},D_{t}^{*},V_{1,t}^{*},V_{2,t}^{*}){\}}_{t=0}^{t=450}$, we infer the parameters related to the vaccination uptake $\theta =[\nu ,n_{v},w_{C},w_{D},w_{V}]$ through sampling the Bayesian posterior distribution with density,


$$\begin{array}{c} p(\theta |𝒟)\propto ℒ(\theta ;𝒟)p(\theta ), \end{array}$$

where ℒ(θ;𝒟) is the likelihood function and p(θ) is the prior probability density. Due to the stochastic nature of the model and the large number of latent states, the likelihood function is intractable, and we rely upon approximate Bayesian computation (ABC) [39,40] to sample the approximate posterior,


$$\begin{array}{c} p(\theta |\rho (𝒟,{𝒟}_{s})\leq \epsilon )\propto \mathbb{P}(\rho (𝒟,{𝒟}_{s})\leq \epsilon |\theta )p(\theta ), \end{array}$$ (5)

where ${𝒟}_{s}$ is a simulated dataset generated from the model with given parameter values, θ, $\rho (𝒟,{𝒟}_{s})$ is a discrepancy metric between the data and simulations, and ϵ is a target discrepancy threshold. For this work, our discrepancy metric between two datasets is the Frobenius norm of the matrix of differences between counts in each dataset (S2 Appendix). To achieve accurate inferences we apply an adaptive sequential Monte Carlo sampler for ABC (SMC-ABC) [41] that produces *N* samples with as small a discrepancy as practically possible. We repeat this inference process for all *M* synthetic datasets, ${𝒟}_{1},{𝒟}_{2},\ldots ,{𝒟}_{M}$, that span the different hesitancy scenarios. Thereby arriving at *M* sets of posterior samples $\{\theta_{1}^{j}{\}}_{j=1}^{j=N},\{\theta_{2}^{j}{\}}_{j=1}^{j=N},$ $\ldots ,\{\theta_{M}^{j}{\}}_{j=1}^{j=N}$. The structure of the resulting posterior samples can be used to investigate if there is information about vaccine hesitancy available in COVID-19 case data and vaccination counts. In all cases the priors are ν~𝒰(0,0.02), $n_{v}\sim 𝒰(0,10)$, $-\log w_{C}\sim 𝒰(3,8)$, $-\log w_{D}\sim 𝒰(3,8)$, and $-\log w_{V}\sim 𝒰(3,8)$.

Model fit is assessed through sampling the within-sample posterior predictive distribution with density,


$$\begin{array}{c} p({𝒟}_{s}|𝒟)=\int_{\Theta }ℒ(\theta ;{𝒟}_{s})p(\theta |𝒟)\;\text{d}\theta . \end{array}$$ (6)

If the model fits well, then we expect the data to lie within the 95% Credible Intervals (95% CrI). Since the models we use to generate synthetic data are all special cases of the full model that is based on (4) and (5), there is no model misspecification error to account for in the inference. However, the posterior predictive check is useful to assess the accuracy of the SMC-ABC sampler with respect to the discrepancy threshold. In practice, model misspecification is important to consider in real applications and we discuss this in the Discussion section.

### Identifiability analysis

In the setting of deterministic models, a parameter is defined as *structurally identifiable* if the mapping from parameter value to a model solution curve is an invertible function. Various methods to analyse structural identifiability for deterministic dynamical systems are available in the literature [42–45]. In contrast, very few methods are available for structural identifiability for stochastic models [24,46]. For simplicity, we derive an equivalent deterministic ordinary differential equation (ODE) approximation (S1 Appendix) to our stochastic model and apply generating series approaches to investigate identifiability [45]. This is appropriate in this setting as the ODE can be considered as an approximation of the mean of the stochastic process as the variance is comparatively negligible due to the large populations in each compartment [24].

In addition to structural identifiability, Bayesian posterior samples provide a way to explore practical identifiability. While frequentist approaches exist for exploring practical identifiability, such as profile likelihoods [47–49], they are challenging to apply for models with intractable likelihood functions [50]. Therefore we adopt a Bayesian approach where practical identifiability is primarily explored via the marginal posterior distributions typically using Markov chain Monte Carlo [24,51] and posterior predictive sampling. Since we are sampling from the joint posterior distribution we automatically have access to samples from the marginal posterior distributions. When marginals themselves demonstrate practical non-identifiability, exploring correlation structures can provide additional insight. In this work, we visually inspect the bivariate marginal structures for each simulation case and Spearman’s rank correlation between pairs of parameters.

## Results

In this section, we present the results of our simulation study. We highlight the key simulation scenarios explored and appropriateness of the model calibration process. We then demonstrate that while individual vaccine uptake parameters may not be uniquely identified from COVID-19 case data, the posterior distributions contain sufficient information to determine the vaccination scenario occurring in the data.

### Simulation scenarios and model calibration

**Fig 4:**
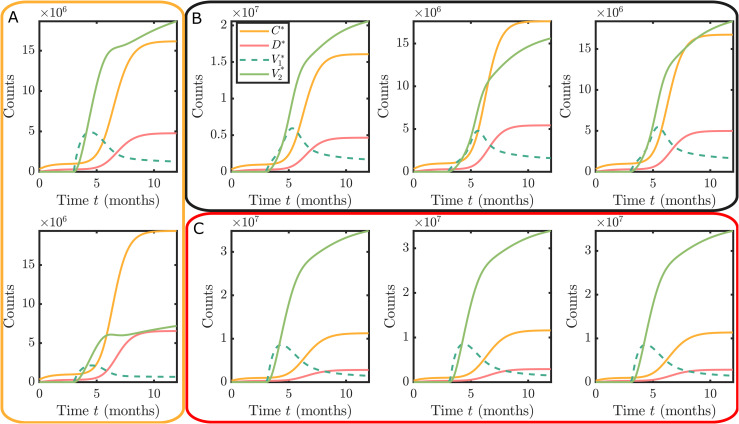
Example synthetic datasets for the three hesitancy scenarios: **(A)** no hesitancy, but smaller maximum vaccination rate than in **(B)** and **(C)**; (B) complacency only with *w*_*C*_ > 0, *w*_*D*_ > 0 and *w*_*V*_ = 0; and (C) complacency and vaccine safety concerns with *w*_*C*_ > 0, *w*_*D*_ > 0 and *w*_*V*_ > 0.

Synthetic datasets were constructed using the model under each vaccination hesitancy behaviour scenario considered in the Methods section. Example synthetic datasets for these three cases are shown in Fig 4.

For each dataset, 𝒟, we apply adaptive SMC-ABC (S2 Appendix) targeting the joint posterior $p(\nu ,n_{v},w_{C},w_{D},w_{V}|𝒟)$. The result is *N* = 1000 samples from the approximate posterior for each synthetic dataset. These samples are used for Monte Carlo integration of posterior probability densities, practical identifiability analysis, and visualisation of parameter correlation structures to investigate the use of COVID-19 case data for identification of vaccine hesitancy patterns.

**Fig 5:**
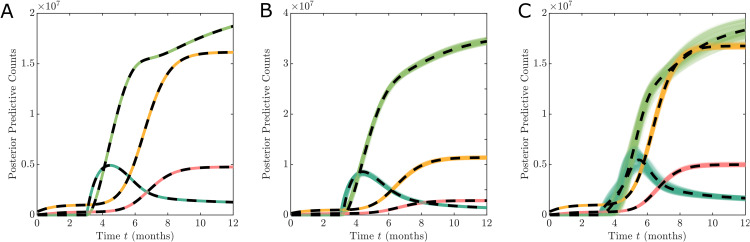
Example posterior predictive simulations (shaded regions) demonstrating model fit for different synthetic datasets (black dashed lines). (A) no hesitancy (true parameters θ=(0.005,4,1,1,1)); (B) complacency only (true parameters $\theta =(0.01,4,5\times 10^{-7},1\times 10^{-6},0)$); and **(C)** Complacency and vaccine safety concerns (true parameters $\theta =(0.01,4,5\times 10^{-7},1\times 10^{-6},1\times 10^{-5})$).

The model fitness is evaluated for each synthetic dataset using within-sample posterior predictive checks. Since the model used for each synthetic dataset is nested within our full model we do not need to account for model misspecification. See Discussion for considerations when misspecification is relevant. The purpose of the posterior predictive check is to ensure the sampler has adapted to a sufficiently small discrepancy threshold for inference. Fig 5 provides example posterior predictive simulations and demonstrates typical model fits obtained from the Bayesian calibration using equivalent convergence criteria for the posterior sampler.

### Identifiability of hesitancy function parameters

We use the GenSSI 2.0 toolkit [43,45] to analyse the structural identifiability of the parameters related to vaccination uptake $\theta =(\nu ,n_{v},w_{C},w_{R},w_{V})$. This approach is applied to a deterministic version of the model. We consider the case when *T*_*v*_ = *T*_*d*_, that is, NPI based restrictions are lifted at the same time as the vaccine roll-out, and the case when $T_{v}\ll T_{d}$, that is, NPI restrictions continue throughout the vaccination roll-out. In both cases we observe only the total reported case numbers, deaths, and vaccinations.

In both cases, all of the vaccine uptake parameters are shown to be locally structurally identifiable. This means that model parameters can be uniquely determined within a subset of parameter space. While this structural identifiability analysis is not directly applicable to the stochastic model, for very large case numbers, the stochastic effects are negligible. Therefore, we conclude that the observation process, if it was continuous, is informative enough to investigate hesitancy behaviours.

**Fig 6:**
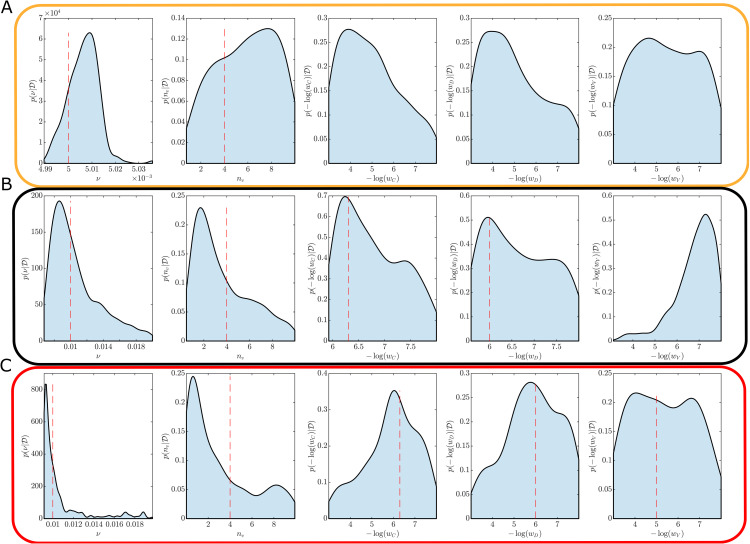
Example marginal posterior densities obtained using synthetic datasets for the three hesitancy scenarios: (A) no hesitancy; (B) complacency only; (C) complacency and vaccine safety concerns. True parameter values are indicated (red dashed lines). Substantial uncertainties in the hesitancy function parameters *n*_*v*_, *w*_*C*_, *w*_*D*_, and *w*_*V*_ indicate practical unidentifiability problems. Cases without a dashed red line indicate cases when the true parameter corresponds to: **(A)** $w_{C},w_{D},w_{V}\to \infty$; and **(B)** $w_{V}\to 0$.

In addition, since our real observation process only records daily counts, we still need to explore practical identifiability as the data are not continuous noise-free observations. The posterior marginal distributions demonstrate practical non-identifiability. This can be observed in the example marginal posterior density plots (Fig 6). Regardless of the synthetic data scenario, the hesitancy function parameters $w_{C},w_{D},$ and *w*_*V*_ shows substantial uncertainty. The only parameter that is well identified is the maximum vaccination rate ν. While the shape of the marginals (particularly the skewness) provide some hints toward recovering the true hesitancy scenario for the respective synthetic data, the differences between the scenarios are subtle.

### Hesitancy inferred through dependency structures

**Fig 7:**
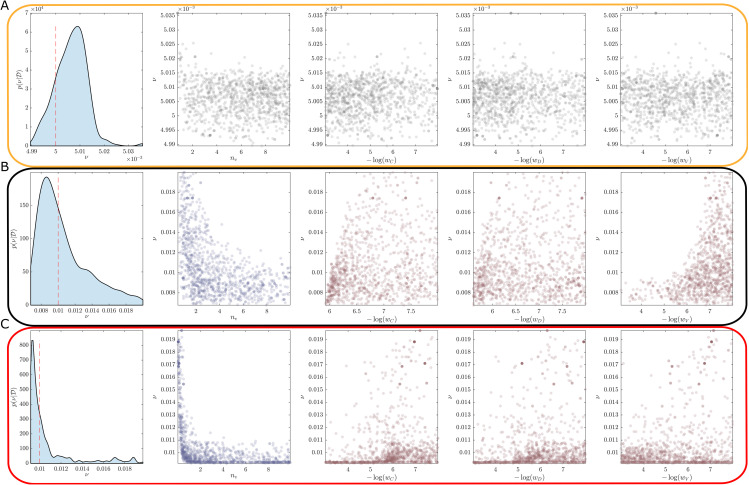
Univariate and bivariate marginal distributions obtained using synthetic datasets for the three hesitancy scenarios: (A) no hesitancy; (B) complacency only; and (C) complacency and vaccine safety concerns. The univariate marginal posterior densities for the maximum vaccination rate, ν, is show with dashed red lines indicating the true values. The scatter plots show the bivariate posterior samples involving ν, from which the pairwise correlations are computed. Plots with significantly positive, significantly negative and statistically insignificant correlations are shown in red, blue and grey respectively.

The marginal posterior distributions indicate that key parameters related to vaccination rates and hesitancy behaviours are practically non-identifiable using COVID-19 case data. However, practical non-identifiability does not preclude the study of the posterior structure to provide insight into the hesitancy behaviours. In particular, the joint posterior distributions contain information about the correlation structure between these parameters. The qualitative form of these correlation structures can be used to uncover the true hesitancy behaviour, albeit without the ability to quantify the actual parameter values reliably. Fig 7 show characteristic example bivariate plot matrices for each of the different vaccination hesitancy scenarios (See S3 Appendix for full bivariate distributions). Throughout, all statistical hypothesis tests are performed at the 0.01 significance level, and correlations refer to Spearman’s rank correlation.

Firstly, by comparing Fig 7(A), that corresponds to synthetic data generated with no hesitancy, with Fig 7(B) and 7(C), that correspond to different hesitancy scenarios, we observe qualitative and quantitative differences in the bivariate posteriors. In the absence of hesitancy in the data, there is no evidence for correlation between ν and the negative log weights, or with the slope parameter *n*_*v*_ as all correlations are statistically insignificant (Fig 7(A)). In contrast, there are statistically significant negative correlations between ν and *n*_*v*_, and statistically significant positive correlations between ν and negative log weights $-\log(w_{C}),-\log(w_{D}),-\log(w_{V})$ (Fig 7(B) and 7(C)). This demonstrates that correlation between the maximum vaccination rate ν with the negative log weight parameters $-\log(w_{C})$, $-\log(w_{D})$, and $-\log(w_{V})$ provide a strong indicator for the presence of hesitancy behaviours, despite univariate marginal parameter estimates being practically non-identifiable.

Second, comparing correlation structures for different cases of hesitancy can inform the components that are dominating behaviour. For example, Fig 7(B) shows the results for a synthetic dataset with only complacency behaviour. Here, the strength of the correlation between ν and $-\log(w_{V})$ is substantially stronger than that of ν with $-\log(w_{C})$ and $-\log(w_{D})$. This indicates the vaccine counts have a much smaller effect on the vaccine uptake than the confirmed cases and deaths. By comparison, a scenario that includes both complacency and vaccine safety concerns (Fig 7(C)) has weaker correlation strengths that are on similar scales. The interpretation here is that increased cases, deaths and vaccine counts all increase the probability of individuals get vaccinated.

Finally, the correlations between the slope *n*_*v*_ parameters also provide some insight, but this information mainly complements the insights obtained through correlations with ν and the other parameters (S3 Appendix). These correlations seem to allow some identification of the dominating complacency drivers between total case numbers or death numbers, however, more work is required to explore this in more detail.

### Summary

Through simulated synthetic dataset scenarios and Bayesian analysis, we demonstrate the ability to identify the presence of vaccine hesitancy at a population level. This is possible despite practical non-identifiability of individual parameters. We achieve this though inspection of the correlation structures in the joint posterior distribution for the parameters relevant to vaccine hesitancy. Further, once vaccine hesitancy behaviours have been identified, it is possible to isolate the specific factors that are driving the hesitancy behaviour. We explore the potential implications and limitations of these results in the Discussion section.

## Discussion

In this work, we explore the feasibility of isolating vaccine hesitancy behaviour and key drivers of this hesitancy using reported case data, such as those available from COVID-19 online dashboards. Using a stochastic epidemic model that accounts for the effects of NPIs and vaccinations over time, we generate synthetic datasets for various hesitancy scenarios with model parameterisation inspired by the early AstraZeneca COVID-19 vaccination roll-out in the United Kingdom. While hesitancy parameters are structurally identifiable, Bayesian analysis reveals that there are practical identifiability challenges for the stochastic model. Despite this, our analysis framework can distinguish the different hesitancy behaviours through inspection of the joint posterior densities and correlation structures. This is potentially a useful tool for future vaccination roll-outs for many diseases aside from COVID-19.

This analysis implies that, in principle, protocols could be developed to quantify the population level effects of hesitancy behaviour during a vaccine roll-out. This could supplement traditional approaches based on survey data to develop information and education campaigns to target hesitancy causes that will have the greatest impact on vaccine uptake. This could be particularly valuable in supplementing survey data when the epidemic situation is rapidly changing as sudden changes in attitudes are difficult to capture with survey data [15]. Furthermore, if reported data is appropriately subdivided, differences in hesitancy behaviour could be assessed for different jurisdictions or vaccine eligibility groups. This could have been applicable during the COVID-19 vaccine roll-out of AstraZeneca when various regulators adjusted the minimum age recommendations in response to concerns around thrombosis and thrombocytopenia syndrome [19,52].

There are several key assumptions within our framework and it is important to highlight them here and discuss the applicability of our methods for real data. Firstly, we consider a spatially homogeneous population in which all individuals have an equal probability of interaction. While we have presented our framework for a large population size, in many realistic settings this assumption will be mainly applicable to smaller spatial scales, such as cities rather than countries. Second, we do not consider an age-structured population nor a staged vaccination roll-out. This does not preclude the utility of our method to be applied to staged programs, provided the reported data is available for the various ages structures and eligibility criterion for each vaccine stage. Third, we assume there is sufficient information on the epidemic and vaccine efficacies to obtain values for all parameters except the vaccination rate and hesitancy parameters. This is not necessarily a major constraint, since substantial epidemiological research will typically proceed vaccination programs and the setting of vaccination roll-out targets, such as vaccine coverage, typically rely on estimates of epidemiological parameters [53–55]. Importantly, as we consider a Bayesian setting, any uncertainties in epidemic parameters or vaccine efficacy can be propagated though our framework through prior specification. Fourth, we only consider a single vaccine with a two dose regime with no hesitancy effects on the second dose. However, the overall approach would still be applicable to multiple vaccine brands by either extending the model to incorporate more vaccination types, or treat vaccination efficacy parameters as an averaged effect. As many data sources do not provide detailed breakdowns of vaccination counts by brand, the latter approach may be preferred. Finally, we assume for simplicity that there is no model misspecification. A key motivation for this was to obtain “best-case” scenarios on what can theoretically be identified about vaccine hesitancy behaviour during a vaccine roll-out. While this assumption will almost certainly not hold in practice, our likelihood-free approach to Bayesian inference (See S2 Appendix) has well-establish properties for natural robustness to misspecification and accounts for model uncertainty [56–58].

In this work we have focused on the identification of hesitancy behaviours for a single well-mixed population. As we have stated in our assumptions, this does not limit the use of our approach to more complex and realistic populations. However, future development of our methods could dramatically expand the rage of possibilities. Various extensions that account for spatial heterogeneity [59], age-structure [60], and seasonality [61] would improve the broad applicability of our methods to more realistic settings. Some extensions, such as spatial heterogeneity, would be challenging to implement using deterministic models, however, these are feasible and completely valid within a stochastic framework. As COVID-19 becomes endemic to a population effects of waning immunity and vaccination booster programs become important [62]. Due to our focus on the initial roll-out of a new vaccine, we have not accounted for these effects here, however, future research should extend our approaches to this case. Finally, vaccine hesitancy could be different for each vaccine brand, therefore a multiple vaccine system could be developed, though it is unclear if the individual hesitancy behaviours will be identifiable in this case.

To implement any of the above mentioned improvements to our modelling framework requires either an extension of the compartment space or the inclusion of hierarchical effects. For models with intractable likelihood functions, as our stochastic model is, such extensions have the potential to dramatically increase the computational cost and inhibit the feasibility of including additional model complexity. However, advanced algorithms for likelihood-free Bayesian computation have the potential to resolve some of these issues. For example, in the hierarchical modelling setting, new computational schemes have been developed in a likelihood-free setting to deal with waning immunity in epidemiological modelling [63]. Furthermore various approaches that exploit surrogate or approximate models have been shown to dramatically improve performance whilst sacrificing minimal accuracy [34,35,64–70]. Finally, state-of-the-art massively parallel computing hardware has potential to complement algorithmic advances to enable real-time analysis [69,71–78].

## Conclusion

Our modelling and computational framework provides a new approach to monitoring vaccine hesitancy using reported case data and vaccination counts that have been widely available during the COVID-19 pandemic. We show that vaccine hesitancy behaviours can be identified from these data provided sufficient information on the epidemic exists preceding the vaccine roll-out. A full Bayesian analysis is performed to be able to identify correlation structure in parameters. It is likely that vaccine hesitancy will continue to be a barrier for vaccine uptake and a major concern for governments during the roll-out of new vaccines in future epidemics. Therefore, our tools that enable the rapid assessment of trends in vaccine hesitancy behaviours within a population can greatly assist public health policy makers and practitioners in addressing public concerns.

## Supporting information

S1 Appendix Stochastic model details. (PDF)

S2 Appendix Approximate Bayesian Analysis. (PDF)

S3 Appendix Additional Results. (PDF)

S1 Code Matlab implementations of models and analysis. (ZIP)
