# Supplementary material for: Bayesian uncertainty quantification to identify population level vaccine hesitancy behaviours
Source: PLoS One. 2026 May 26;21(5):e0349499. doi: 10.1371/journal.pone.0349499 (PMC13210240; doi:10.1371/journal.pone.0349499)
Supplement: S1 Appendix — (PDF) [file pone.0349499.s001.pdf]

Supporting Information

S1 Appendix. Stochastic model details. In this section, we present the mathematical details of the base stochastic epidemiological model and the extension including vaccinations and hesitancy behaviour.

Base Model Formulation

We consider a stochastic susceptible-exposed-infectious-recovered (SEIR) compartmental model based upon the work of [1] and [2]. In this framework, a population of size P is divided into four main epidemiological compartments: susceptible (S), exposed (E), infectious (I) and recovered (R). Since we cannot observe exactly the true number of active infectious individuals, these four components are considered unobservable or latent states. Infectious individuals will be identified as active confirmed cases (A^*) at rate $\gamma > 0$. These active confirmed cases will transition to reported deaths (D^*) or recoveries (R^*) at rates $\delta > 0$ and $\rho > 0$ respectively. These three compartments are observable and correspond to reported COVID-19 data available from online dashboards and repositories such as Johns Hopkins University [3] https://coronavirus.jhu.edu/ or Our World in Data [4] https://ourworldindata.org/coronavirus. It should be noted that most data sources only reliably record the cumulative case numbers, $C^* = A^* + R^* + D^*$, and reported deaths, D^*. In addition, the reported C^* will be an underestimate of the true number of cumulative cases since infectious individuals can also recover without being reported as a confirmed case. In our model this transition occurs with rate $\eta > 0$.

In the SEIR model of the latent compartments, exposed individuals E will become infectious at rate $\beta > 0$ and those that are infectious can recover at rate $\eta > 0$. The population R represents cases in which the virus has run its course and the individual has received immunity without contributing to the COVID-19 case numbers; such is the case with asymptomatic cases that did not get tested. We do not explicitly model the possibility of a unreported death due to COVID-19, rather we absorbed this into the R compartment, that is, R is a removed category. The novelty of the approach by [1] and [2] is the transmission mechanism in which a susceptible individual will be exposed with hazard rate $[\alpha_0 + \alpha g(\cdot))]I/P$, where $\alpha_0 > 0$ is the residual transmission rate, $\alpha > 0$ is the transmission rate parameter that can be affected by changes in interaction behaviours of the population. These changes are modelled using a response function, $g(\cdot)$, that depends on the observables A^*, D^*, R^* and on time t. The feedback loop induced by the response function models the way the population changes behaviour in response external information such as public health advice, NPIs, or media reports. Similar approaches have also been considered for other diseases, such as influenza and HIV [5, 6]. Figure 1 shows a schematic of the base model including the latent variables, S, E, I and R, and the feedback mechanism through $g(\cdot)$.

Let $\mathbf{X}_t = [S_t, E_t, I_t, R_t, A_t^*, D_t^*, R_t^*]^{\mathrm{T}}$ denote the state of the population at time $t \in (0, T]$ with $t = 0$ at the initial condition and $t = T$ at the last day of the time-series. Similarly, $\mathbf{Z}_t = [A_t^*, D_t^*, R_t^*]^{\mathrm{T}}$ refers to the vector of observables. Under the assumption of a spatially homogeneous population and exponential waiting time between events, then we arrive at a discrete-state Markov process that can be expressed in the Kurtz random time-change representation,

$$\mathbf{X}_t = \mathbf{X}_0 + \sum_{j=1}^{M} Y_j \left(\int_0^t h_j(\mathbf{X}_s, s)\,\mathrm{d}s \right) \boldsymbol{\nu}_j, \quad (1)$$

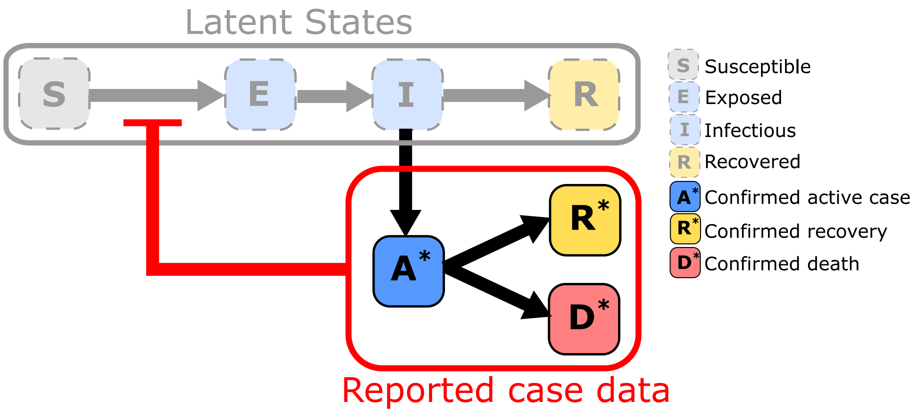

Fig 1. Schematic of the base epidemiological model. States are marked as labelled boxes and arrows indicate state transitions. The model consists of an unobservable (latent) SEIR model with the reported case data arising through the transition from I to A^*. The transmission is inhibited by a response function that depends on the observed reported case data.

where M is the number of transition events, $\mathcal{E}_1, \mathcal{E}_2, \ldots, \mathcal{E}_M$, $h_j(\cdot)$ and $\boldsymbol{\nu}_j$ are, respectively, the hazard function and state change for event $\mathcal{E}_j$, and $Y_j(\cdot)$ is an inhomogeneous Poisson process with mean $\int_0^t h_j(\mathbf{X}_s, s)\,\mathrm{d}s$. For this model, we have $M = 6$ and

$$\begin{aligned}
&\mathcal{E}_1 : S \to E, && h_1(\mathbf{X}_t, t) = [\alpha_0 + \alpha g(\mathbf{Z}_t, t)]\frac{I_t S_t}{P}, && \boldsymbol{\nu}_1 = [-1, 1, 0, 0, 0, 0, 0]^{\mathrm{T}},\\
&\mathcal{E}_2 : E \to I, && h_2(\mathbf{X}_t, t) = \beta E_t, && \boldsymbol{\nu}_2 = [0, -1, 1, 0, 0, 0, 0]^{\mathrm{T}},\\
&\mathcal{E}_3 : I \to R, && h_3(\mathbf{X}_t, t) = \eta I_t, && \boldsymbol{\nu}_3 = [0, 0, -1, 1, 0, 0, 0]^{\mathrm{T}},\\
&\mathcal{E}_4 : I \to A^*, && h_4(\mathbf{X}_t, t) = \gamma I_t, && \boldsymbol{\nu}_4 = [0, 0, -1, 0, 1, 0, 0]^{\mathrm{T}},\\
&\mathcal{E}_5 : A^* \to D^*, && h_5(\mathbf{X}_t, t) = \delta A_t^*, && \boldsymbol{\nu}_5 = [0, 0, 0, 0, -1, 1, 0]^{\mathrm{T}},\\
&\mathcal{E}_6 : A^* \to R^*, && h_6(\mathbf{X}_t, t) = \rho A_t^*, && \boldsymbol{\nu}_6 = [0, 0, 0, 0, -1, 0, 1]^{\mathrm{T}}.
\end{aligned}$$

Here, $\mathbf{Z}_t = [A_t^*, D_t^*, R_t^*]^{\mathrm{T}}$ refers to the vector of observables. Such a system can be simulated exactly using the Gillespie direct method or approximately using a time discretisation such as the τ-leaping scheme [7]. In this work all stochastic simulations are performed using a τ-leaping scheme with $\tau = 1$ day which is sufficiently accurate for our modelling purposes [1, 2].

To initialise simulations, typically only C_0^* and D_0^* are available from data, we therefore denote two additional parameters $\kappa \geq 0$ and $\zeta \in [0, 1]$ to initialise the other compartments. $\kappa > 0$ denotes the number of latent infections per active confirmed case and ζ is the proportion of alive cases that are currently active. Thus we arrive at initial states, $A_0^* = \lceil \zeta(C_0^* - D_0^*) \rceil$, $R_0^* = \lfloor (1 - \zeta)(C_0^* - D_0^*) \rfloor$, $I_0^* = \lceil \kappa A_0^* \rceil$, $E_0 = 2I_0$, $R_0 = 0$, and $S_0 = P - (E_0 + I_0 + C_0^* + D_0^*)$.

Many different forms could be considered to describe different restriction strategies based on NPIs. For example in [1], a lockdown strategy based on trigger threshold is implemented with

$$g(\mathbf{Z}_t^*) = \frac{1}{1 + (w_A A_t^*)^n}, \tag{2}$$

where $w_A > 0$ is a weight that affects the point in which the lockdown reaches 50% efficacy (that is $g(1/w_A) = 1/2$) and $n > 0$ controls the rate at which the lockdown is introduced or eased (Figure 2(E)). That is, a larger weight corresponds to a rapid

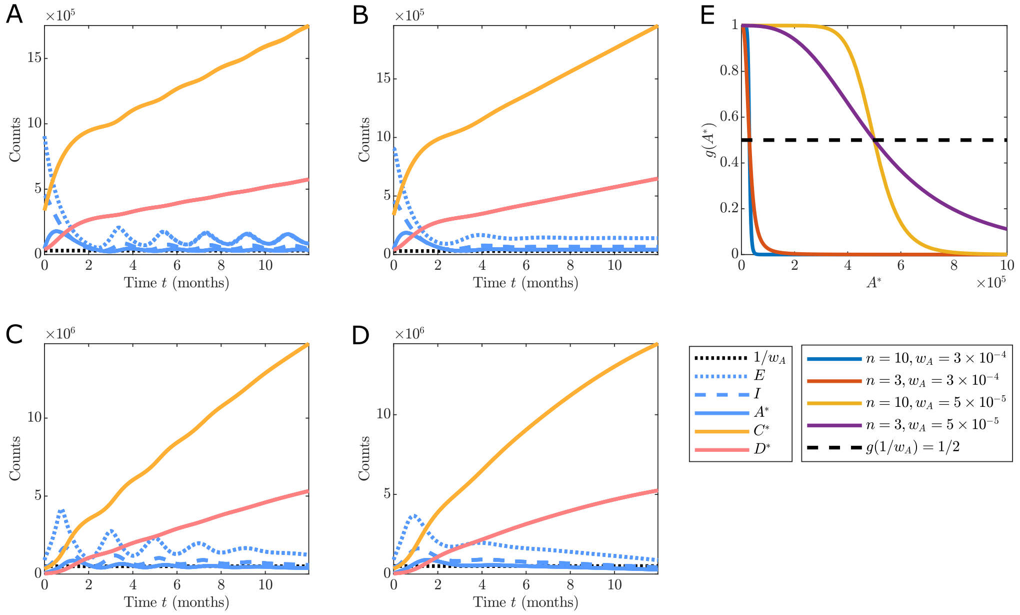

Fig 2. Realisations of the base epidemiological model for different configurations of the response function $g(A^*) = 1/(1 + (w_A A^*)^n)$ with: (A) $n = 10$ and $w_A = 3 \times 10^{-4}$; (B) $n = 3$ and $w_A = 3 \times 10^{-4}$; (C) $n = 10$ and $w_A = 5 \times 10^{-5}$; and (D) $n = 3$ and $w_A = 5 \times 10^{-5}$. The shape of the response function in each case is shown in (E). Simulations are initialised based on the UK data on 1st September 2020 with $C_0^* = 337,798$, $D_0^* = 41,551$ and model parameters are $\alpha_0 = 0.05$ [days^{-1}], $\alpha = 0.4$ [days^{-1}], $\beta = 0.07$ [days^{-1}], $\gamma = 0.05$ [days^{-1}], $\delta = 0.03$ [days^{-1}], $\rho = 0.05$, $\eta = 0.1$ [days^{-1}], $\kappa = 10$, and $\zeta = 0.15$. The τ-leaping approximate stochastic simulation algorithm is used with time-step $\tau = 1$ [days].

response (Figure 2(E) blue and red curves) and a smaller weight to a delayed response (Figure 2(E) yellow and purple curves). The slop parameter $n > 0$ relate to how sudden the introduction (resp. relaxation) of the response with larger n being a greater rate of introduction (Compare the yellow curve ,$n = 10$, with the purple curve, $n = 3$, in Figure 2(E)). The choices for w_A and n have a substantial impact on the dynamics of the outbreak as shown in Figure 2(A)–(D). Figure 2 represents hypothetical scenarios initialised using confirmed case and death data for the United Kingdom on 1st September 2020 with $C_0^* = 337,798$ and $D_0^* = 41,551$. Unsurprisingly, we observe an early response leads to fewer cases and deaths than a delayed response (Compare Figure 2(A) with Figure 2(C) and Figure 2(B) with Figure 2 (D)). A sudden response leads to more frequent, higher peak, short duration epidemic waves, whereas a gradual response leads to infrequent, lower peak, long duration waves (Compare Figure 2(A) with Figure 2(B) and Figure 2(C) with Figure 2 (D)).

Multiphase behaviour in the response function can be introduced to simulate changes in intervention strategy over time. To do this we allow the response function to depend on time as well as on the observables. For example, consider a lockdown strategy following Equation (2) that is enacted over the interval $t \in [0, T_d]$, before easing all restrictions for $t > T_d$. This could be implemented with the response function,

$$g(\mathbf{Z}_t^*, t) = \frac{1}{1 + (\mathbb{1}_{[0,T_d]}(t)\, w_A A_t^*)^n}, \tag{3}$$

where $\mathbb{1}_{[0,T_d]}(t)$ denotes an indicator function with $\mathbb{1}_{[0,T_d]}(t) = 1$ for $t \in [0, T_d]$ and $\mathbb{1}_{[0,T_d]}(t) = 0$ otherwise. In Figure 3(A)–(D), we use the same initial conditions as in

Figure 2 and $T_d = 92$ corresponds to the 2nd of December 2020 when the second UK lockdown ended. As expected, we observe that regardless of the response applied for $t \leq T_d$, in the absence of vaccination, complete removal of restrictions for $t > T_d$ leads to the same cumulative case and death counts.

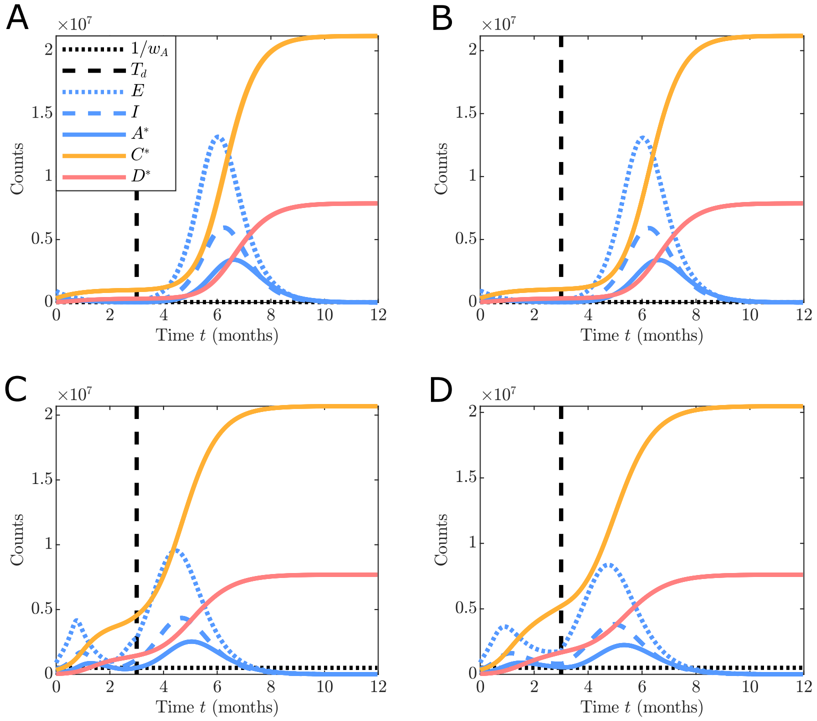

Fig 3. Realisations of the base epidemiological model for different configurations of the multiphase response function $g(A^*, t) = 1/(1 + (\mathbb{1}_{[0,T_d]}(t)\, w_A A^*)^n)$ with: (A) $n = 10$ and $w_A = 3 \times 10^{-4}$; (B) $n = 3$ and $w_A = 3 \times 10^{-4}$; (C) $n = 10$ and $w_A = 5 \times 10^{-5}$; and (D) $n = 3$ and $w_A = 5 \times 10^{-5}$. Simulations are initialised based on the UK data on 1st September 2020 with $C_0^* = 337,798$, $D_0^* = 41,551$ and model parameters are $\alpha_0 = 0.05\,[\text{days}^{-1}]$, $\alpha = 0.4\,[\text{days}^{-1}]$, $\beta = 0.07\,[\text{days}^{-1}]$, $\gamma = 0.05\,[\text{days}^{-1}]$, $\delta = 0.03\,[\text{days}^{-1}]$, $\rho = 0.05$, $\eta = 0.1\,[\text{days}^{-1}]$, $\kappa = 10$, $\zeta = 0.15$, and $T_d = 92\,[\text{days}]$ corresponding to the 2nd of December 2020. The τ-leaping approximate stochastic simulation algorithm is used with time-step $\tau = 1\,[\text{days}]$.

Full Model Formulation

We implement our model as a stochastic compartmental epidemiological model consisting of 21 the compartments, that consist of 7 main compartment types (i.e., S (susceptible), E exposed, I (infectious), R recovered, A^* (confirmed active case), R^* (confirmed case recovery) and D^* (confirmed case fatality)) with each occurring in one of three levels of vaccinations status (e.g., for susceptible compartment: S_u unvaccinated, S_1, vaccinated (1st dose), S_2 vaccinated (2nd dose)). We refer to the main manuscript for parameter and compartment definitions (Sections 2.1).

There are 28 transition events, $\mathcal{E}_1, \mathcal{E}_2, \ldots, \mathcal{E}_{28}$, between the 21 compartments. These transition events and their respective hazard functions, assuming a well mixed

population P, are

$$
\begin{aligned}
&\mathcal{E}_1: \; S_u + I_u \to I_u + E_u, h_1(\mathbf{X}_t) = (\alpha_0 + \alpha g(\mathbf{Z}_t, t))\, \frac{I_{u,t} S_{u,t}}{P},\\
&\mathcal{E}_2: \; S_u + I_1 \to I_1 + E_u, \, h_2(\mathbf{X}_t) = \alpha_v^1 \,(\alpha_0 + \alpha g(\mathbf{Z}_t, t))\, \frac{I_{1,t} S_{u,t}}{P},\\
&\mathcal{E}_3: \; S_u + I_2 \to I_2 + E_u, \, h_3(\mathbf{X}_t) = \alpha_v^2 \,(\alpha_0 + \alpha g(\mathbf{Z}_t, t))\, \frac{I_{2,t} S_{u,t}}{P},\\
&\mathcal{E}_4: \; E_u \to I_u, \qquad h_4(\mathbf{X}_t) = \beta E_u,\\
&\mathcal{E}_5: \; I_u \to R_u, \qquad h_5(\mathbf{X}_t) = \eta I_u,\\
&\mathcal{E}_6: \; I_u \to A_u^*, \qquad h_6(\mathbf{X}_t) = \gamma I_u,\\
&\mathcal{E}_7: \; A_u^* \to R_u^*, \qquad h_7(\mathbf{X}_t) = \rho A_u^*,\\
&\mathcal{E}_8: \; A_u^* \to D_u^*, \qquad h_8(\mathbf{X}_t) = \delta A_u^*,\\
&\mathcal{E}_9: \; S_u \to S_1, \qquad h_9(\mathbf{X}_t) = \nu h(\mathbf{Z}_t, t) S_u,\\
&\mathcal{E}_{10}: R_u \to R_1, \qquad h_{10}(\mathbf{X}_t) = \nu h(\mathbf{Z}_t, t) R_u,\\
&\mathcal{E}_{11}: S_1 + I_u \to I_u + E_1, \, h_{11}(\mathbf{X}_t) = \alpha_v^1 \,(\alpha_0 + \alpha g(\mathbf{Z}_t, t))\, \frac{I_{u,t} S_{1,t}}{P},\\
&\mathcal{E}_{12}: S_1 + I_1 \to I_1 + E_1, \, h_{12}(\mathbf{X}_t) = (\alpha_v^1)^2 \,(\alpha_0 + \alpha g(\mathbf{Z}_t, t))\, \frac{I_{1,t} S_{1,t}}{P},\\
&\mathcal{E}_{13}: S_1 + I_2 \to I_2 + E_1, \, h_{13}(\mathbf{X}_t) = \alpha_v^1 \alpha_v^2 \,(\alpha_0 + \alpha g(\mathbf{Z}_t, t))\, \frac{I_{2,t} S_{1,t}}{P},\\
&\mathcal{E}_{14}: E_1 \to I_1, \qquad h_{14}(\mathbf{X}_t) = \beta E_1,\\
&\mathcal{E}_{15}: I_1 \to R_1, \qquad h_{15}(\mathbf{X}_t) = \eta I_1,\\
&\mathcal{E}_{16}: I_1 \to A_1^*, \qquad h_{16}(\mathbf{X}_t) = \gamma I_1,\\
&\mathcal{E}_{17}: A_1^* \to R_1^*, \qquad h_{17}(\mathbf{X}_t) = \rho A_1^*,\\
&\mathcal{E}_{18}: A_1^* \to D_1^*, \qquad h_{18}(\mathbf{X}_t) = \delta_v^1 \delta A_1^*,
\end{aligned}
$$

$$
\begin{aligned}
&\mathcal{E}_{19}: S_1 \to S_2, \qquad h_{19}(\mathbf{X}_t) = \omega S_1,\\
&\mathcal{E}_{20}: R_1 \to R_2, \qquad h_{20}(\mathbf{X}_t) = \omega R_1,\\
&\mathcal{E}_{21}: S_2 + I_u \to I_u + E_2, h_{21}(\mathbf{X}_t) = \alpha_v^2 \,(\alpha_0 + \alpha g(\mathbf{Z}_t, t))\, \frac{I_{u,t} S_{2,t}}{P},\\
&\mathcal{E}_{22}: S_2 + I_1 \to I_2 + E_1, \, h_{22}(\mathbf{X}_t) = \alpha_v^1 \alpha_v^2 \,(\alpha_0 + \alpha g(\mathbf{Z}_t, t))\, \frac{I_{1,t} S_{2,t}}{P},\\
&\mathcal{E}_{23}: S_2 + I_2 \to I_2 + E_2, \, h_{23}(\mathbf{X}_t) = (\alpha_v^2)^2 \,(\alpha_0 + \alpha g(\mathbf{Z}_t, t))\, \frac{I_{2,t} S_{2,t}}{P},\\
&\mathcal{E}_{24}: E_2 \to I_2, \qquad h_{24}(\mathbf{X}_t) = \beta E_2,\\
&\mathcal{E}_{25}: I_2 \to R_2, \qquad h_{25}(\mathbf{X}_t) = \eta I_2,\\
&\mathcal{E}_{26}: I_2 \to A_2^*, \qquad h_{26}(\mathbf{X}_t) = \gamma I_2,\\
&\mathcal{E}_{27}: A_2^* \to R_2^*, \qquad h_{27}(\mathbf{X}_t) = \rho A_2^*,\\
&\mathcal{E}_{28}: A_2^* \to D_2^*, \qquad h_{28}(\mathbf{X}_t) = \delta_v^2 \delta A_2^*.
\end{aligned}
$$

The respective state change vectors $\boldsymbol{\nu}_1, \boldsymbol{\nu}_2, \ldots, \boldsymbol{\nu}_{28}$ can be obtained in a straightforward manner based on the ordering of compartments in the full system state vector is $\mathbf{X}_t = [S_{u,t}, E_{u,t}, I_{u,t}, R_{u,t}, A_{u,t}^*, R_{u,t}^*, D_{u,t}^*, S_{1,t}, \ldots, D_{1,t}^*, \, S_{2,t}, \ldots, D_{2,t}^*]$. The observable

data are $\mathbf{Z}_t = [C_t^*, D_t^*, V_{1,t}^*, V_{2,t}^*]$ with

$$\begin{aligned}
C_t^* &= A_{u,t}^* + A_{1,t}^* + A_{2,t}^* + R_{u,t}^* + R_{1,t}^* + R_{2,t}^* \\
D_t^* &= D_{u,t}^* + D_{1,t}^* + D_{2,t}^* \\
V_{1,t}^* &= S_{1,t} + E_{1,t} + I_{1,t} + R_{1,t} + A_{1,t}^* + R_{1,t}^* + D_{1,t}^* \\
V_{2,t}^* &= S_{2,t} + E_{2,t} + I_{2,t} + R_{2,t} + A_{2,t}^* + R_{2,t}^* + D_{2,t}^*.
\end{aligned}$$

The response function $g(\mathbf{Z}_t, t)$ and hesitancy effect function $h(\mathbf{Z}_t, t)$ are given by,

$$\begin{aligned}
g(\mathbf{Z}_t, t) &= \frac{1}{1 + (\mathbb{1}_{[0,T_d]}(t)\, w_A \zeta C_t^*)^n}, \\
h(\mathbf{Z}_t, t) &= \frac{(w_C C^* + w_D D^* + w_V V_2^*)^{n_v}}{1 + (w_C C^* + w_D D^* + w_V V_2^*)^{n_v}} \mathbb{1}_{[T_v,\infty)}(t).
\end{aligned}$$

These correspond to Equations (2) and (6) in the main manuscript.

Stochastic Simulation

While exact realisations of this process can be generated using event-based simulation [8, 9], this is prohibitive within an approximate Bayesian computational setting with large population sizes and event numbers. Therefore, we apply a first order approximation to the integral over the interval $[t, t+\tau)$ to obtain the tau-leaping approximation [7],

$$\mathbf{X}_{t+\tau} = \mathbf{X}_t + \sum_{j=1}^{28} Y_j \boldsymbol{\nu}_j + \mathcal{O}(\tau),$$

where $Y_j \sim \text{Poisson}\,(h_j(\mathbf{X}_t)\tau)$ counts the number of times event j occurs in the interval $[t, t+\tau)$. Simulations proceed as per Gillespie [**?**]. For our simulations we use $\tau = 1$ (days), and initial condition $\mathbf{X}_0 = [P - (3\kappa\zeta + 1)C_0^* - D_0^*, 2\kappa\zeta C_0^*, \kappa C_0^*, 0, \zeta C_0^*, (1-\zeta)C_0^*, D_0^*, \mathbf{0}]^{\mathrm{T}}$.

ODE Model for Structural Identifiability Analysis

We derived a deterministic version of our epidemiological model, representing the mean system evolution. This enabled the application of techniques form the `GenSSI 2.0` toolkit for structural identifiability analysis. The model is a nonlinear system of 23 ordinary differential equations.

The virus transmission model, includin regulatory behaviour thought the function $g(A^*)$, and vaccination (with hesitancy effect $h(C^*, D^*, V_2^*)$) is given by,

$$\begin{aligned}
\frac{\mathrm{d}S_u}{\mathrm{d}t} &= -(\alpha_0 + \alpha g(A^*))\frac{I_u + \sum_{j=1}^{j=2} \alpha_v^j I_v^j}{P} S_u - \nu h(C^*, D^*, V_2^*) S_u \\
\frac{\mathrm{d}E_u}{\mathrm{d}t} &= (\alpha_0 + \alpha g(A^*))\frac{I_u + \sum_{j=1}^{j=2} \alpha_v^j I_v^j}{P} S_u - \beta E_u \\
\frac{\mathrm{d}I_u}{\mathrm{d}t} &= \beta E_u - (\eta + \gamma) I_u, \quad \frac{\mathrm{d}R_u}{\mathrm{d}t} = \eta I_u - \nu h(C^*, D^*, V_2^*) R_u \\
\frac{\mathrm{d}A_u^*}{\mathrm{d}t} &= \gamma I_u - (\delta + \rho) A_u^*, \quad \frac{\mathrm{d}R_u^*}{\mathrm{d}t} = \rho A_u^*, \quad \frac{\mathrm{d}D_u^*}{\mathrm{d}t} = \delta A_u^*,
\end{aligned} \tag{4}$$

where, $g(A^*) = \dfrac{1}{1 + (w_A A^*)^n}$ and $h(C^*, D^*, V_2^*) = \dfrac{(w_C C^* + w_D D^* + w_V V_2^*)^{n_v}}{1 + (w_C C^* + w_D D^* + w_V V_2^*)^{n_v}}$.

Then the two dose vaccination processes is given by,

$$\begin{aligned}\frac{\mathrm{d}S_v^1}{\mathrm{d}t} &= \nu h(C^*, D^*, V_2^*)S_u - \alpha_v^1(\alpha_0 + \alpha g(A^*))\frac{I_u + \sum_{j=1}^{j=2}\alpha_v^j I_v^j}{P}S_v^1 - \omega S_v^1 \\ \frac{\mathrm{d}S_v^2}{\mathrm{d}t} &= \omega S_v^1 - \alpha_v^2(\alpha_0 + \alpha g(A^*))\frac{I_u + \sum_{j=1}^{j=2}\alpha_v^j I_v^j}{P}S_v^1 \\ \frac{\mathrm{d}R_v^1}{\mathrm{d}t} &= \nu h(C^*, D^*, V_2^*)R_u - \omega R_v^1, \quad \frac{\mathrm{d}R_v^2}{\mathrm{d}t} = \omega R_v^1.\end{aligned} \tag{5}$$

Finally, the effects on vaccination in disease progression, spreading and mortality is given by,

$$\begin{aligned}\frac{\mathrm{d}E_v^i}{\mathrm{d}t} &= \alpha_v^i(\alpha_0 + \alpha g(A^*))\frac{I_u + \sum_{j=1}^{j=2}\alpha_v^j I_v^j}{P}S_v^i - \beta E_v^i \\ \frac{\mathrm{d}I_v^i}{\mathrm{d}t} &= \beta E_v^i - (\eta + \gamma)I_v^i, \quad \frac{\mathrm{d}R_v^i}{\mathrm{d}t} = \eta I_v^i - \nu h(C^*, D^*, V_2^*)R_v^i \\ \frac{\mathrm{d}A_v^{i,*}}{\mathrm{d}t} &= \gamma I_v^i - (\delta_v^i\delta + \rho)A_v^{i,*}, \quad \frac{\mathrm{d}R_v^{i,*}}{\mathrm{d}t} = \rho A_v^{i,*}, \quad \frac{\mathrm{d}D_v^{i,*}}{\mathrm{d}t} = \delta_v^i \delta A_v^{i,*}.\end{aligned} \tag{6}$$

Observable states are,

$$\begin{aligned}&A^* = A_v^* + \sum_{j=1}^{2} A_v^{i,*}, \quad R^* = R_v^* + \sum_{j=1}^{2} R_v^{i,*}, \quad D^* = D_v^* + \sum_{j=1}^{2} D_v^{i,*} \\ &V_2 = S_v^2 + E_v^2 + I_v^2 + R_v^2 + A_v^{2,*} + R_v^{2,*} + D_v^{2,*}, \quad \text{and } C^* = A^* + R^* + D^*.\end{aligned} \tag{7}$$

References

1. Warne DJ, Ebert A, Drovandi C, Hu W, Mira A, Mengersen K. Hindsight is 2020 vision: a characterisation of the global response to the COVID-19 pandemic. BMC Public Health. 2020;20(1). doi:10.1186/s12889-020-09972-z.

2. Le TM, Raynal L, Talbot O, Hambridge H, Drovandi C, Mira A, et al. Framework for assessing and easing global COVID-19 travel restrictions. Scientific Reports. 2022;12(1). doi:10.1038/s41598-022-10678-y.

3. Johns Hopkins University. Coronavirus resource centre; 2020. Available from: `https://coronavirus.jhu.edu/`.

4. Mathieu E, Ritchie H, Rodés-Guirao L, Appel C, Giattino C, Hasell J, et al.. Coronavirus Pandemic (COVID-19); 2020. Available from: `https://ourworldindata.org/coronavirus`.

5. Collinson S, Heffernan JM. Modelling the effects of media during an influenza epidemic. BMC Public Health. 2014;14(1). doi:10.1186/1471-2458-14-376.

6. Teng TRY, Lara-Tuprio EPD, Macalalag JMR. An HIV/AIDS epidemic model with media coverage, vertical transmission and time delays. In: AIP Conference Proceedings. AIP Publishing; 2019.

7. Gillespie DT. Approximate accelerated stochastic simulation of chemically reacting systems. The Journal of Chemical Physics. 2001;115(4):1716–1733. doi:10.1063/1.1378322.

8. Gibson MA, Bruck J. Efficient Exact Stochastic Simulation of Chemical Systems with Many Species and Many Channels. The Journal of Physical Chemistry A. 2000;104(9):1876–1889. doi:10.1021/jp993732q.

9. Gillespie DT. Exact stochastic simulation of coupled chemical reactions. The Journal of Physical Chemistry. 1977;81(25):2340–2361. doi:10.1021/j100540a008.
