# Supplementary material for: Bayesian uncertainty quantification to identify population level vaccine hesitancy behaviours
Source: PLoS One. 2026 May 26;21(5):e0349499. doi: 10.1371/journal.pone.0349499 (PMC13210240; doi:10.1371/journal.pone.0349499)
Supplement: S2 Appendix — (PDF) [file pone.0349499.s002.pdf]

Supporting Information

S2 Appendix. Approximate Bayesian analysis. We apply Bayesian inference to quantify uncertainty in the model parameters associated vaccine hesitancy effects, $\boldsymbol{\theta} = [\nu, n_n, w_C, w_D, w_V]$, for each synthetic dataset i that represents the structure of data sets available from Johns Hopkins University or Our World in Data dashboards, that is, $\mathcal{D}_i = [\{C^*_{t,i}, D^*_{t,i}, V^*_{1,t,i}, V^*_{2,t,i}\}_{T\geq t\geq 0}]$ where T is the total number of days available of data. The task is to sample the posterior distribution with probability density given by Bayes' Theorem,

$$p(\boldsymbol{\theta} \mid \mathcal{D}_i) = \frac{p(\mathcal{D}_i \mid \boldsymbol{\theta})p(\boldsymbol{\theta})}{p(\mathcal{D}_i)},$$

where $p(\boldsymbol{\theta})$ is the prior, $p(\mathcal{D}_i \mid \boldsymbol{\theta})$ is the likelihood and $p(\mathcal{D}_i)$ is the evidence. For the remainder of this section, we omit the country index i for notational convenience.

Since the full model state vector is only partially observable, the data model is no longer Markovian and is therefore computationally intractable. To deal with this likelihood intractability, we apply approximate Bayesian computation (ABC) [1–3], that samples from an approximation to the posterior for each country,

$$\begin{aligned} p(\boldsymbol{\theta} \mid \mathcal{D}) \approx p(\boldsymbol{\theta} \mid \rho(\mathcal{D}, \mathcal{D}_s) \leq \epsilon) &\propto \mathbb{P}(\rho(\mathcal{D}, \mathcal{D}_s) \leq \epsilon \mid \boldsymbol{\theta})p(\boldsymbol{\theta}) \\ &= p(\boldsymbol{\theta}) \int \mathbb{1}_{(0,\epsilon]}\left(\rho(\mathcal{D}, \mathcal{D}_s)\right) s(\mathcal{D}_s \mid \boldsymbol{\theta})\,\mathrm{d}\mathcal{D}_s, \end{aligned}$$

where $\mathcal{D}$ is the synthetic data that will align to one of the hesitancy scenarios scenario, $\mathcal{D}_s \sim s(\cdot \mid \boldsymbol{\theta})$ is simulated data from the full model, $\rho(\mathcal{D}, \mathcal{D}_s)$ is a discrepancy metric, ϵ is the discrepancy threshold and $\mathbb{1}_{(0,\epsilon]}\left(\rho(\mathcal{D}, \mathcal{D}_s)\right) = 1$ if $\rho(\mathcal{D}, \mathcal{D}_s) \leq \epsilon$, and $\mathbb{1}_{(0,\epsilon]}\left(\rho(\mathcal{D}, \mathcal{D}_s)\right) = 0$ otherwise. For our implementation, we apply the discrepancy metric,

$$\rho(\mathcal{D}, \mathcal{D}_s) = \left(\sum_{t=1}^{T} (C^*_t - C^*_{t,s})^2 + (D^*_t - D^*_{t,s})^2 + (V^*_{1,t} - V^*_{1,t,s})^2 + (V^*_{2,t} - V^*_{2,t,s})^2)\right)^{1/2}$$

where $\mathcal{D} = [\{A^*_t, D^*_t, V^*_{1,t}, V^*_{2,t}\}_{t\geq 0}]$ is the synthetic data for a given scenario and $\mathcal{D}_s = [\{A^*_{t,s}, D^*_{t,s}, V^*_{1,t,s}, V^*_{2,t,s}\}_{t\geq 0}]$ is simulated data form the full model.

Sequential Monte Carlo sampling

We apply a sequential Monte Carlo (SMC) scheme [**?**, **?**] to move an initial set of N_p samples from the prior through a sequence of ABC approximations defined by a decreasing sequence of T discrepancy thresholds, $\epsilon_1 > \epsilon_2 > \cdots > \epsilon_T = \epsilon$. Our particular implementation (Algorithm 1), based on the work of Drovandi and Pettit [**?**], adaptively selects the acceptance thresholds and utilises MCMC steps using tuned Gaussian random walk proposals. For all model calibrations we apply adaptive SMC with $N_p = 1000$ particles, tuning parameters $c = 0.01$, $a = 0.5$ and terminate sampling when the MCMC acceptance probability p_{acc} drops below $p_{\min} = 0.01$. The acceptance probability is estimated using $R_{\text{trial}} = 5$ initial MCMC iterations per particle.

Algorithm 1 Adaptive SMC sampler for approxmate Bayesian computation

Initialise $N_a = aN_p$, $N_\ell = N_p - N_a$
for $j \in [1, 2, \ldots, N_p]$ **do**
    Sample prior, $\boldsymbol{\theta}^* \sim p(\cdot)$ and simulate model, $\mathcal{D}_s \sim s(\cdot \mid \boldsymbol{\theta}^*)$;
    Set $\rho_j \leftarrow \rho(\mathcal{D}, \mathcal{D}_s)$;
end for
repeat
    Sort particles $\{(\boldsymbol{\theta}_j, \rho_j)\}_{j=1}^{N_p}$, such that $\rho_j \leq \rho_{j+1}$ for all $j \in [1, 2, \ldots, N_p - 1]$;
    Remove particles $\{(\boldsymbol{\theta}_j, \rho_j)\}_{j=N_\ell+1}^{N_p}$ an set $\epsilon \leftarrow \rho_{N_\ell}$;
    Resample particles $\{\boldsymbol{\theta}_j\}_{j=N_\ell+1}^{N_p}$ from $\{(\boldsymbol{\theta}_j)\}_{j=1}^{N_\ell}$ with replacement;
    Estimate sample covariance, $\hat{\boldsymbol{\Sigma}}$, of particles $\{\boldsymbol{\theta}_j\}_{j=1}^{N_p}$.
    Adapt proposal kernel $q(\mathbf{u} \mid \mathbf{v}) = \phi\left(\mathbf{u}; \mathbf{v}, \dfrac{2.38^2}{\dim(\boldsymbol{\theta})}\hat{\boldsymbol{\Sigma}}\right)$, where $\phi(\cdot; \boldsymbol{\mu}, \boldsymbol{\Sigma})$ is a multivariate Gaussian density function and $\dim(\boldsymbol{\theta})$ is the number of parameters;
    Set $p_{\text{acc}} \leftarrow 0$;
    for $j \in [N_\ell + 1, N_\ell + 2, \ldots, N_p]$ **do**
        for $k \in [1, 2, \ldots, R_{\text{trial}}]$ **do**
            Generate proposal, $\boldsymbol{\theta}^* \sim q(\cdot \mid \boldsymbol{\theta}_j)$ and sample $u \sim \mathcal{U}(0,1)$;
            if $u \leq \min\left(1, \dfrac{p(\boldsymbol{\theta}^*)q(\boldsymbol{\theta}_j \mid \boldsymbol{\theta}^*)}{p(\boldsymbol{\theta}_j)q(\boldsymbol{\theta}^* \mid \boldsymbol{\theta}_j)}\right)$ **then**
                Simulate model $\mathcal{D}_s \sim s(\cdot \mid \boldsymbol{\theta}^*)$;
                if $\rho(\mathcal{D}_i, \mathcal{D}_s) \leq \epsilon$ **then**
                    Set $\boldsymbol{\theta}_j \leftarrow \boldsymbol{\theta}^*$, $\rho_j \leftarrow \rho(\mathcal{D}_i, \mathcal{D}_s)$, and $p_{\text{acc}} \leftarrow p_{\text{acc}} + (R_{trial}N_a)^{-1}$;
                end if
            end if
        end for
    end for
    Set $R \leftarrow \log c / \log(1 - p_{\text{acc}})$;
    for $j \in [N_\ell + 1, N_\ell + 2, \ldots, N_p]$ **do**
        for $k \in [1, 2, \ldots, R - R_{\text{trial}}]$ **do**
            Generate proposal, $\boldsymbol{\theta}^* \sim q(\cdot \mid \boldsymbol{\theta}_j)$ and sample $u \sim \mathcal{U}(0,1)$;
            if $u \leq \min\left(1, \dfrac{p(\boldsymbol{\theta}^*)q(\boldsymbol{\theta}_j \mid \boldsymbol{\theta}^*)}{p(\boldsymbol{\theta}_j)q(\boldsymbol{\theta}^* \mid \boldsymbol{\theta}_j)}\right)$ **then**
                Simulate model $\mathcal{D}_s \sim s(\cdot \mid \boldsymbol{\theta}^*)$;
                if $\rho(\mathcal{D}_i, \mathcal{D}_s) \leq \epsilon$ **then**
                    Set $\boldsymbol{\theta}_j \leftarrow \boldsymbol{\theta}^*$, $\rho_j \leftarrow \rho(\mathcal{D}_i, \mathcal{D}_s)$, and $p_{\text{acc}} \leftarrow p_{\text{acc}} + (RN_a)^{-1}$;
                end if
            end if
        end for
    end for
until $p_{\text{acc}} < p_{\min}$

References

1. Sisson SA, Fan Y, Beaumont MA, editors. Handbook of Approximate Bayesian Computation. Chapman and Hall/CRC; 2018.

2. Sunnåker M, Busetto AG, Numminen E, Corander J, Foll M, Dessimoz C. Approximate Bayesian Computation. PLoS Computational Biology. 2013;9(1):e1002803. doi:10.1371/journal.pcbi.1002803.

3. Warne DJ, Baker RE, Simpson MJ. Simulation and inference algorithms for stochastic biochemical reaction networks: from basic concepts to state-of-the-art. Journal of The Royal Society Interface. 2019;16(151):20180943. doi:10.1098/rsif.2018.0943.
