# Supplementary material for: Bayesian uncertainty quantification to identify population level vaccine hesitancy behaviours
Source: PLoS One. 2026 May 26;21(5):e0349499. doi: 10.1371/journal.pone.0349499 (PMC13210240; doi:10.1371/journal.pone.0349499)
Supplement: S3 Appendix — (PDF) [file pone.0349499.s003.pdf]

Supporting Information

S3 Appendix. Additional Results. Figures 1–8 show equivalent Bayesian analyses results for other synthetic data set configurations. The patterns related to bivariate marginal posterior correlation structures are consistent with those discussed in the main manuscript.

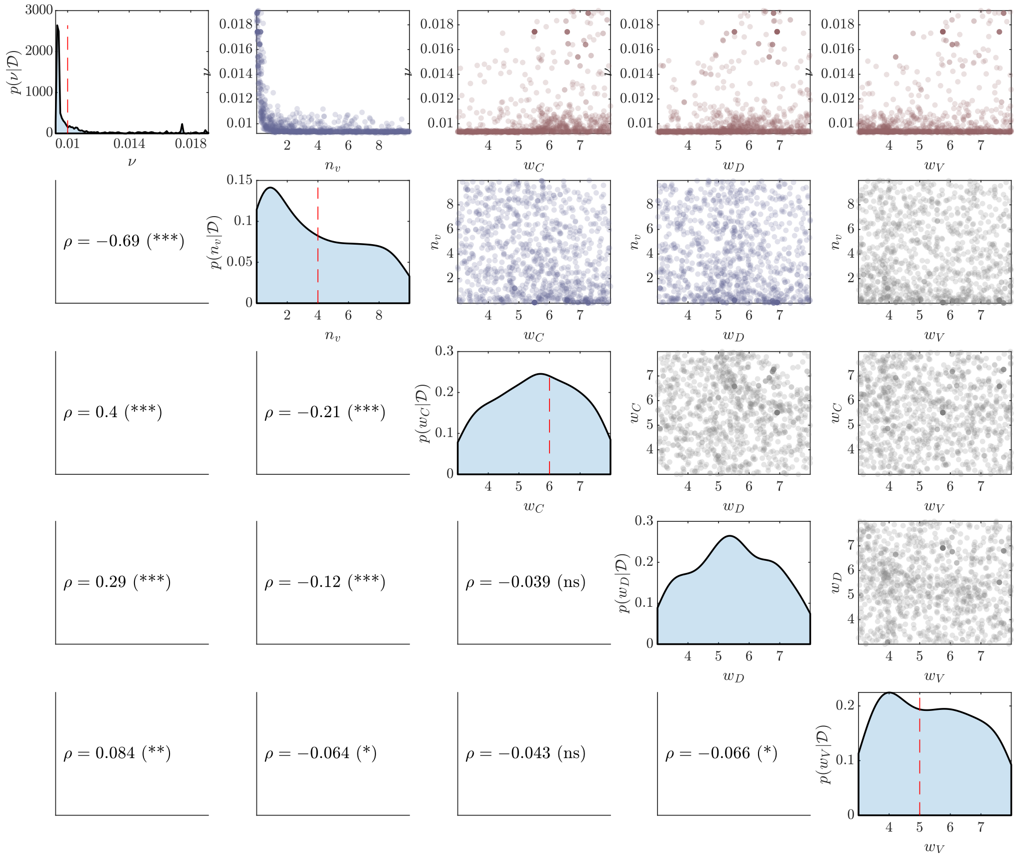

Fig 1. Example posterior samples for the simulation scenario with $\nu = 0.01$, $n_v = 4$, $w_C = 10^{-6}$, $w_D = 0$ and $w_V = 2 \times 10^{-6}$. The main diagonals show marginal posterior densities with dashed red lines indicating the true values. Marginals without a dashed red line indicates the true parameter corresponds to $w_C, w_D, w_V \to \infty$ or $w_C, w_D, w_V \to 0$. The upper off-diagonal plots show the bivariate posterior samples, from which the pairwise correlations are computed. Plots with significantly positive, significantly negative and statistically insignificant correlations are shown in red, blue and grey respectively. The lower off-diagonal plots show the Spearman rank correlation and statistical significance level.

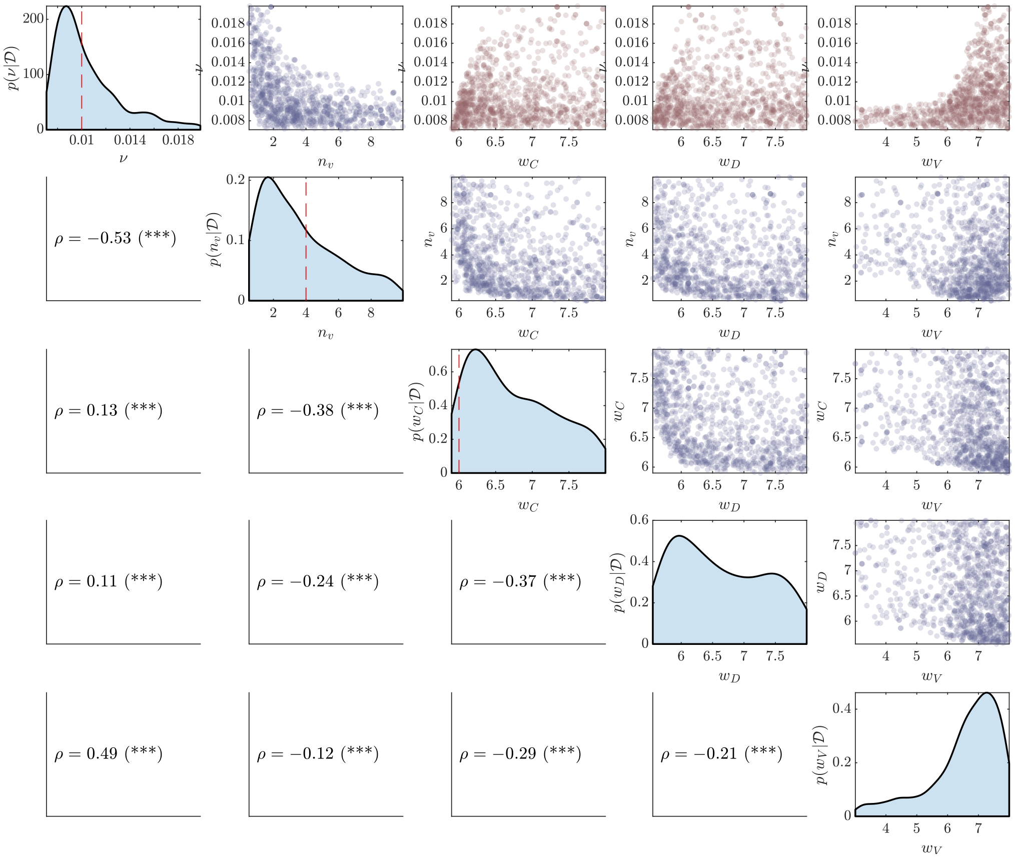

Fig 2. Example posterior samples for the simulation scenario with $\nu = 0.01$, $n_v = 4$, $w_C = 10^{-6}$, $w_D = 0$ and $w_V = 0$. The main diagonals show marginal posterior densities with dashed red lines indicating the true values. Marginals without a dashed red line indicates the true parameter corresponds to $w_C, w_D, w_V \to \infty$ or $w_C, w_D, w_V \to 0$. The upper off-diagonal plots show the bivariate posterior samples, from which the pairwise correlations are computed. Plots with significantly positive, significantly negative and statistically insignificant correlations are shown in red, blue and grey respectively. The lower off-diagonal plots show the Spearman rank correlation and statistical significance level.

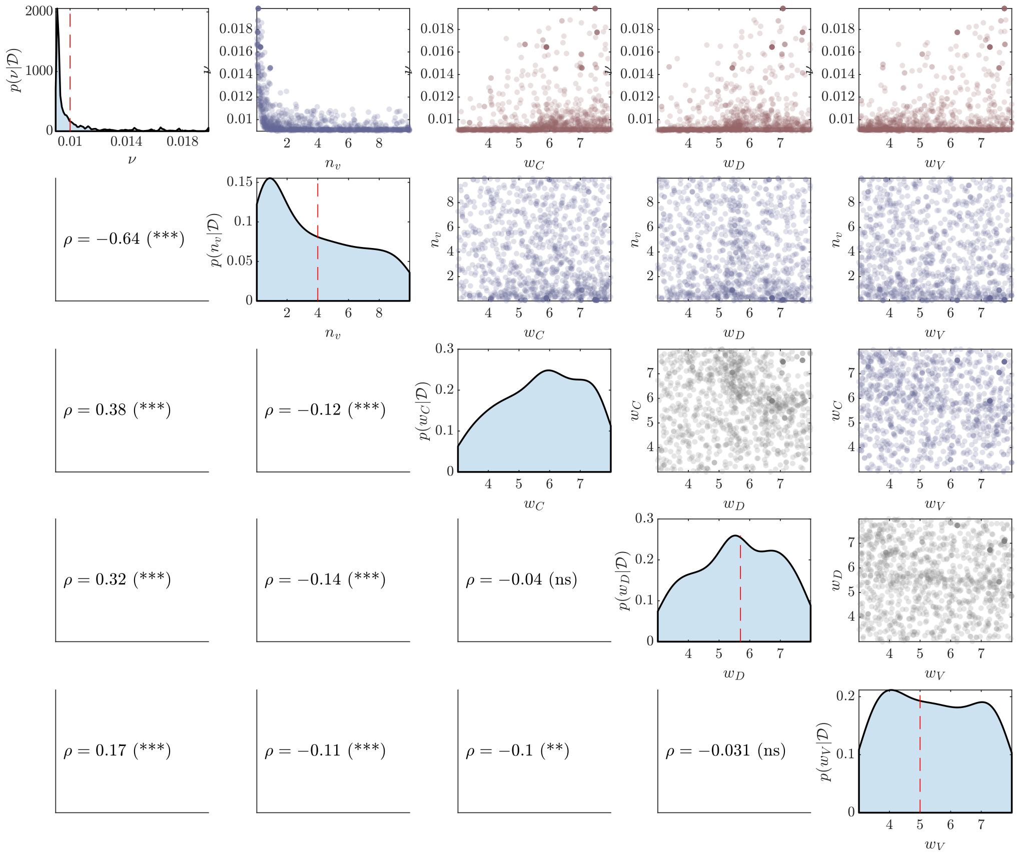

Fig 3. Example posterior samples for the simulation scenario with $\nu = 0.01$, $n_v = 4$, $w_C = 0$, $w_D = 2 \times 10^{-6}$ and $w_V = \times 10^{-5}$. The main diagonals show marginal posterior densities with dashed red lines indicating the true values. Marginals without a dashed red line indicates the true parameter corresponds to $w_C, w_D, w_V \to \infty$ or $w_C, w_D, w_V \to 0$. The upper off-diagonal plots show the bivariate posterior samples, from which the pairwise correlations are computed. Plots with significantly positive, significantly negative and statistically insignificant correlations are shown in red, blue and grey respectively. The lower off-diagonal plots show the Spearman rank correlation and statistical significance level.

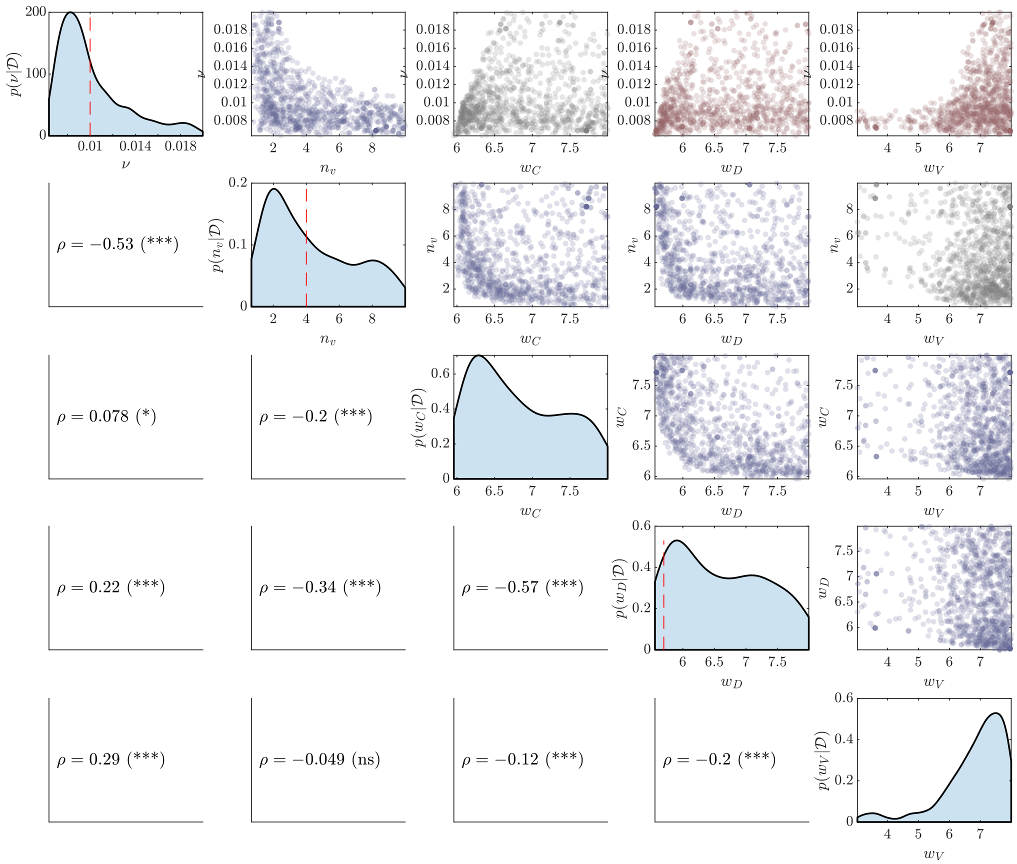

Fig 4. Example posterior samples for the simulation scenario with $\nu = 0.01$, $n_v = 4$, $w_C = 0$, $w_D = 2 \times 10^{-6}$ and $w_V = 0$. The main diagonals show marginal posterior densities with dashed red lines indicating the true values. Marginals without a dashed red line indicates the true parameter corresponds to $w_C, w_D, w_V \to \infty$ or $w_C, w_D, w_V \to 0$. The upper off-diagonal plots show the bivariate posterior samples, from which the pairwise correlations are computed. Plots with significantly positive, significantly negative and statistically insignificant correlations are shown in red, blue and grey respectively. The lower off-diagonal plots show the Spearman rank correlation and statistical significance level.

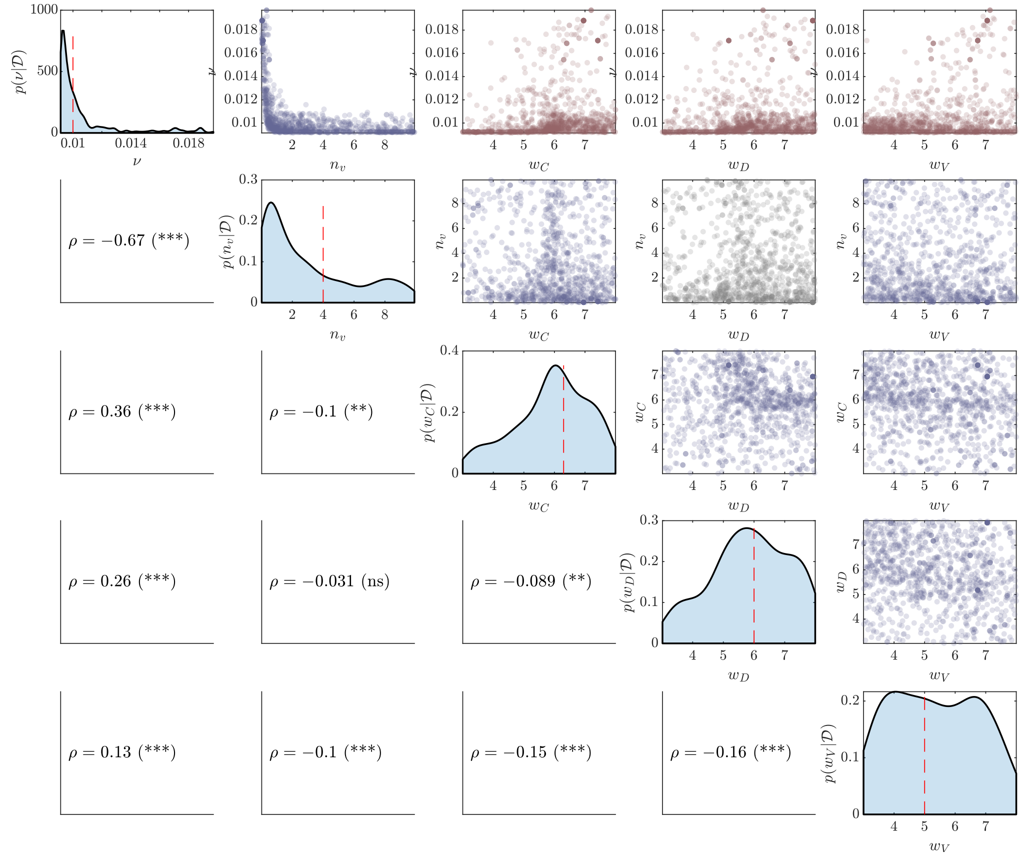

Fig 5. Example posterior samples for the simulation scenario with $\nu = 0.01$, $n_v = 4$, $w_C = 5 \times 10^{-7}$, $w_D = \times 10^{-6}$ and $w_V = 10^{-5}$. The main diagonals show marginal posterior densities with dashed red lines indicating the true values. Marginals without a dashed red line indicates the true parameter corresponds to $w_C, w_D, w_V \to \infty$ or $w_C, w_D, w_V \to 0$. The upper off-diagonal plots show the bivariate posterior samples, from which the pairwise correlations are computed. Plots with significantly positive, significantly negative and statistically insignificant correlations are shown in red, blue and grey respectively. The lower off-diagonal plots show the Spearman rank correlation and statistical significance level.

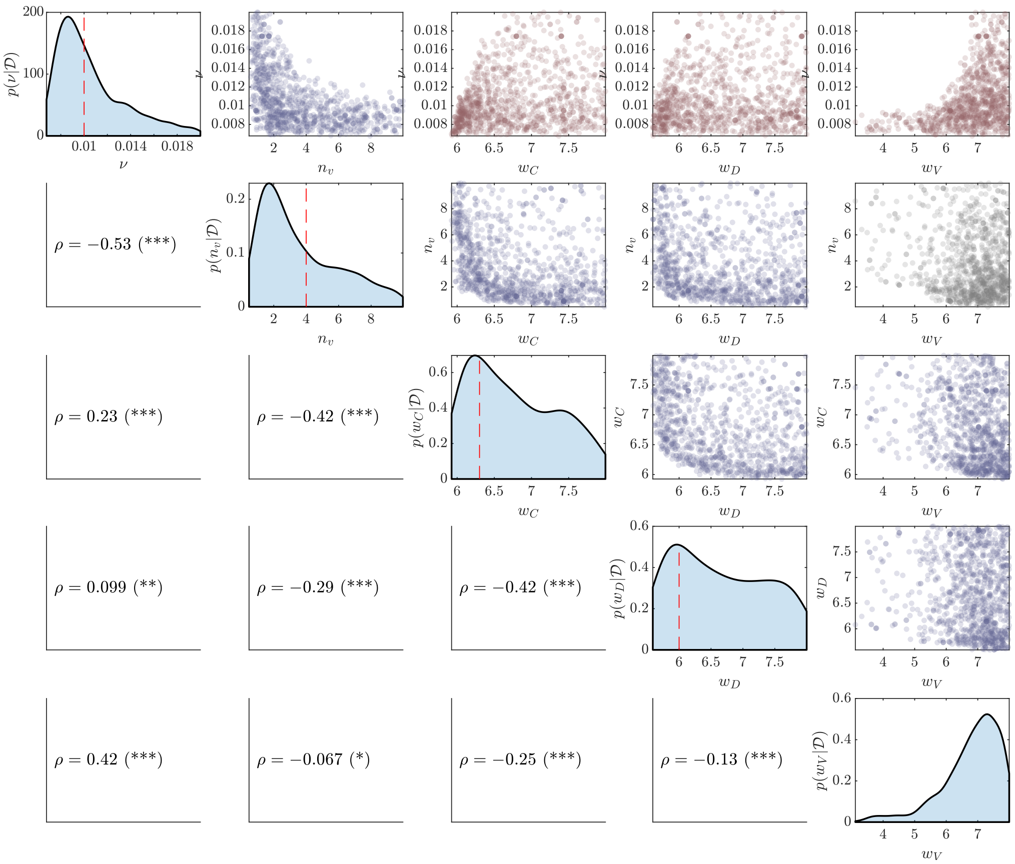

Fig 6. Example posterior samples for the simulation scenario with $\nu = 0.01$, $n_v = 4$, $w_C = 5 \times 10^{-7}$, $w_D = \times 10^{-6}$ and $w_V = 0$. The main diagonals show marginal posterior densities with dashed red lines indicating the true values. Marginals without a dashed red line indicates the true parameter corresponds to $w_C, w_D, w_V \to \infty$ or $w_C, w_D, w_V \to 0$. The upper off-diagonal plots show the bivariate posterior samples, from which the pairwise correlations are computed. Plots with significantly positive, significantly negative and statistically insignificant correlations are shown in red, blue and grey respectively. The lower off-diagonal plots show the Spearman rank correlation and statistical significance level.

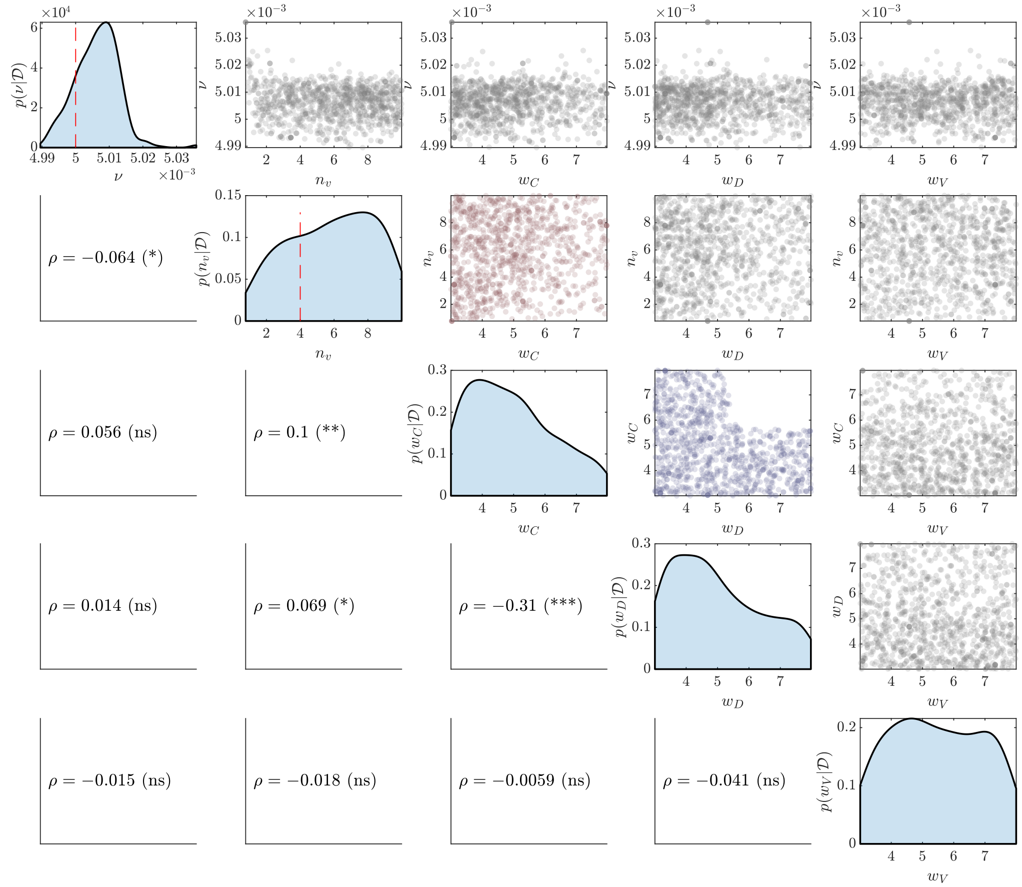

Fig 7. Example posterior samples for the simulation scenario with $\nu = 0.005$, $n_v = 4$, $w_C to \infty$, $w_D = 0$ and $w_V = 0$. The main diagonals show marginal posterior densities with dashed red lines indicating the true values. Marginals without a dashed red line indicates the true parameter corresponds to $w_C, w_D, w_V \to \infty$ or $w_C, w_D, w_V \to 0$. The upper off-diagonal plots show the bivariate posterior samples, from which the pairwise correlations are computed. Plots with significantly positive, significantly negative and statistically insignificant correlations are shown in red, blue and grey respectively. The lower off-diagonal plots show the Spearman rank correlation and statistical significance level.

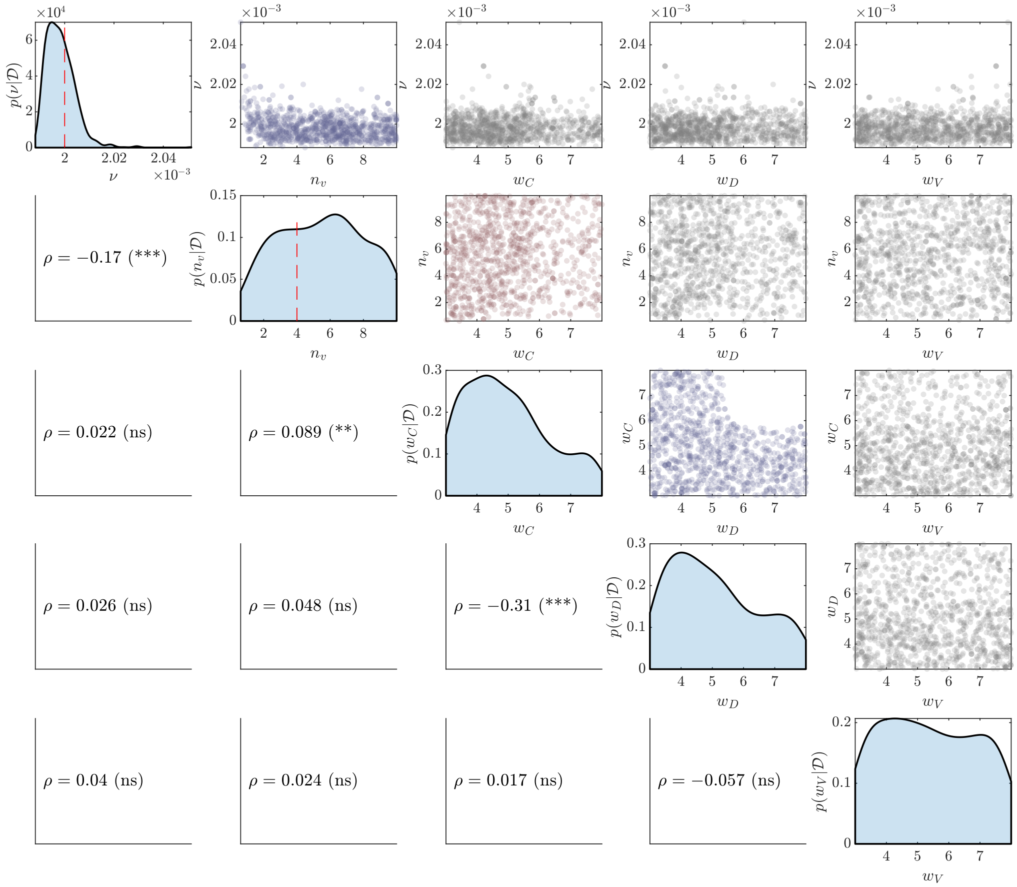

Fig 8. Example posterior samples for the simulation scenario with $\nu = 0.002$, $n_v = 4$, $w_C to \infty$, $w_D = 0$ and $w_V = 0$. The main diagonals show marginal posterior densities with dashed red lines indicating the true values. Marginals without a dashed red line indicates the true parameter corresponds to $w_C, w_D, w_V \to \infty$ or $w_C, w_D, w_V \to 0$. The upper off-diagonal plots show the bivariate posterior samples, from which the pairwise correlations are computed. Plots with significantly positive, significantly negative and statistically insignificant correlations are shown in red, blue and grey respectively. The lower off-diagonal plots show the Spearman rank correlation and statistical significance level.
